# Hexokinase gene *OsHXK1* positively regulates leaf senescence in rice

**DOI:** 10.1186/s12870-021-03343-5

**Published:** 2021-12-08

**Authors:** Shaoyan Zheng, Jingqin Lu, Di Yu, Jing Li, Hai Zhou, Dagang Jiang, Zhenlan Liu, Chuxiong Zhuang

**Affiliations:** 1 State Key Laboratory for Conservation and Utilization of Subtropical Agro-Bioresources, South China Agricultural University, Guangzhou, 510642 China; 2 Guangdong Laboratory for Lingnan Modern Agriculture, Guangzhou, 510642 China

**Keywords:** *OsHXK1*, Rice, Leaf senescence, ROS, Glucose

## Abstract

**Background:**

Leaf senescence is a highly complex and meticulous regulatory process, and the disruption of any factor involved in leaf senescence might lead to premature or delayed leaf senescence and thus result in reduced or increased crop yields. Despite sincere efforts by scientists, there remain many unsolved problems related to the regulatory factors and molecular mechanisms of leaf senescence.

**Results:**

This study successfully revealed that *OsHXK1* was highly expressed in senescent leaves of rice. The upregulation of *OsHXK1* led to premature senescence of rice leaves, a decreased level of chlorophyll, and damage to the chloroplast structure. The overexpression of *OsHXK1* resulted in increases in glucose and ROS levels and produced programmed cell death (PCD) signals earlier at the booting stage. Further analysis showed that expression level of the respiratory burst oxidase homolog (RBOH) genes and *OsGLO1* were increased in *OsHXK1*-overexpressing plants at the booting stage.

**Conclusions:**

Overall, the outcomes of this study suggested that *OsHXK1* could act as a positive regulator of rice leaf senescence by mediating glucose accumulation and inducing an increase in ROS.

**Supplementary Information:**

The online version contains supplementary material available at 10.1186/s12870-021-03343-5.

## Background

Leaf senescence is a very complicated and programmed process involving protein synthesis, degradation, transportation, and other metabolic changes. Leaf senescence is a characteristic that is influenced by many environmental factors, such as abiotic and biological stress [1]. The leaf is an important functional organ of rice and includes the onset of senescence, senescence decline, and the end of senescence. During the period from heading to fruiting of rice and other crops, early leaf senescence will reduce the accumulation of dry matter during grain filling and limit the improvement in crop yield and quality [2]. In rice, factors affecting the initial stage of leaf senescence mainly include the plant endogenous hormones, temperature, light, nutrient elements, and the coordination of sink-source relationship [3]. Abscisic acid and ethylene can accelerate the senescence of leaves in vitro, while cytokinin can delay the senescence of leaves by inhibiting the activities of ribonuclease and protease and delaying the degradation of nucleic acids, proteins, and chloroplasts. The main indexes to measure senescence are chlorophyll content, protein content, and reactive oxygen species (ROS) level [4]. Genes that regulate rice senescence also include transcription factors, NAC (NAM, ATAF1/2 and CUC2)/WRKY/MYB family transcription factors, kinases, proteases, lipases, and ribonucleases. The cloning and functional elucidation of most genes related to green retention accelerated progress in rice senescence research [5–8]. Among those genes, *OsNAP* is one of the NAC transcription factors that affects the expression of nutrient remobilization- and senescence-related genes to regulate chlorophyll degradation, leading to leaf senescence via the abscisic acid (ABA) pathway [9].

SGR (stay-green-rice) was the first cloned gene that is upregulated in senescent leaves of rice, which affects the degradation rate of chlorophyll in leaves [10]. *Osl2*, a pyruvate-dependent GABA transaminase, is upregulated to enhance enzyme activities in senescing rice leaves [11]. *Osh69* plays an important role in the degradation of chloroplast galactolipids during leaf senescence [12]. *OsDos* (*Oryza sativa* delay of the onset of senescence) is involved in delaying leaf senescence in rice by integrating developmental cues into the jasmonic acid (JA) pathway [13]. *OsNYC1* (NONYELLOW COLORING1) encodes a chlorophyll b reductase and plays a role in this process by effectively inhibiting the degradation of chlorophyll [14]. *OsNYC3* (Non-Yellow Coloring 3) and *OsNOL* (NYC1-LIKE) are responsible for chlorophyll degradation in rice [15, 16]. Common changes caused by these genes include the degradation of chlorophyll, accumulation of reactive oxygen species, carbon, and nitrogen imbalances, and responses to hormones in a coordinated manner at the cellular and organismal levels. The identification of premature senescence genes is of great significance to explore the mechanism of premature senescence and improve the yield of rice.

In old leaves, sugar accumulation can induce leaf senescence due to the induction of higher utilization of carbon than nitrogen. Sugar-induced leaf senescence is particularly important under low-nitrogen utilization conditions and plays a crucial role in the process of sensing light signals [1, 12, 17]. Previous studies have demonstrated that sugar starvation can affect the expression of genes related to senescence [18–21]. Analyses of sugar treatments have shown that 2% glucose combined with a low nitrogen supply can induce leaf senescence and the expression of a set of genes in Arabidopsis, such as senescence-specific gene SAGs. The expression of *SAG12* exhibits high senescence specificity, and during this process, hexose is highly accumulated in senescent leaves of *Arabidopsis* at later stages of development [18–21]. These results indicate that intracellular sugar levels can mediate leaf senescence.

In plants, hexokinase (HXK) is a key component of the glycolysis pathway. HXK is bifunctional and can catalyze the phosphorylation of two hexose molecules to form hexose-6-phosphate for input into the glycolysis pathway and has important functions in sugar signal transduction and sensing [22–26]. According to previous studies, hexokinases can associate with subcellular compartments, including mitochondria, the Golgi complex in the cytoplasm, and chloroplasts, which suggests that hexokinase may have many different intracellular functions [27]. Hexokinase links sugar signals with environmental factors and hormone signals and thus controls photosynthesis, growth and development, flowering, and senescence in plants [28].

Many hexokinases have been found in plants, among which HXK in *Arabidopsis thaliana* has been studied extensively. The *Arabidopsis* genome encodes six hexokinase members. A study of the *Arabidopsis HXK1* mutant *gin2* revealed the involvement of the sugar signaling pathway in the regulation of leaf senescence; hexokinase, as a sugar sensor, actively participates in the transmission of sugar signals during leaf senescence [29]. *AtHXK1* can sense glucose signals and transgenic plants in the *gin2* mutant background expressing catalytically inactive *AtHXK1* mutant alleles sensing multiple signaling processes. *AtHXK1* can play a role in the processes of leaf senescence and sugar signal transmission even under the interaction between light and hormones; overexpression of *AtHXK1* can inhibit plant growth and promote leaf senescence [29, 30], which suggests that the sensory and catalytic functions of *AtHXK1* in *Arabidopsis* plants may be uncoupled.

The rice genome has ten hexokinase genes. Previous studies have revealed that the protein encoded by *OsHXK4* is located in the chloroplast membrane and functions in fatty acid and starch synthesis processes [28]. The proteins encoded by *OsHXK5*/*OsHXK*6 exert dual targeting to localize to the nucleus and mitochondria. Overexpression of *OsHXK5*/*OsHXK*6 leads to arrested growth, and combined with sugar treatment, this overexpression inhibits expression of the photosynthesis gene *RbcS*. Therefore, it has been hypothesized that *OsHXK5* and *OsHXK*6 have the function of glucose sensing, similar to *AtHXK1* [31]. In contrast, *OsHXK*7 is mainly involved in glycolysis or cytoplasmic metabolism, such as sucrose biosynthesis, by scavenging free hexose in the cytoplasm [28]. *OsHXK10* is specifically expressed during pollen development in rice. Rice plants with decreased expression of *OsHXK10* exhibit blocked anther dehiscence, decreased pollen fertility, and decreased seed germination activity [32]. In our previous study, we reported that *OsHXK1* can regulate the initiation of PCD signaling in the anther tapetum and regulate rice yield and photosynthesis [33, 34], but the relationship of *OsHXK1* with leaf senescence and metabolic regulation in rice remains to be confirmed.

In this study, we identified *OsHXK1* as a key regulator in the development of rice leaf senescence. This gene positively regulated the leaf senescence process by regulating ROS accumulation in rice leaves. This research fundamentally uncovered the internal constraints of leaf senescence in rice to provide a key basis for high-quality and antiaging rice variety breeding.

## Results

### *OsHXK1* is highly expressed in senescent leaves

As described previously, *OsHXK1* (Os07g0446800) regulates the initiation of tapetal programmed cell death (PCD) in rice anthers and regulates rice photosynthesis [33, 34]. Interestingly, we found that transgenic plants with increased *OsHXK1* expression exhibited withered and yellow leaf tips and margins toward the base of the leaf compared with wild-type plants at the flowering and filling stages. Therefore, we continued to reveal the function of *OsHXK1* in leaf development.

**Fig. 1:**
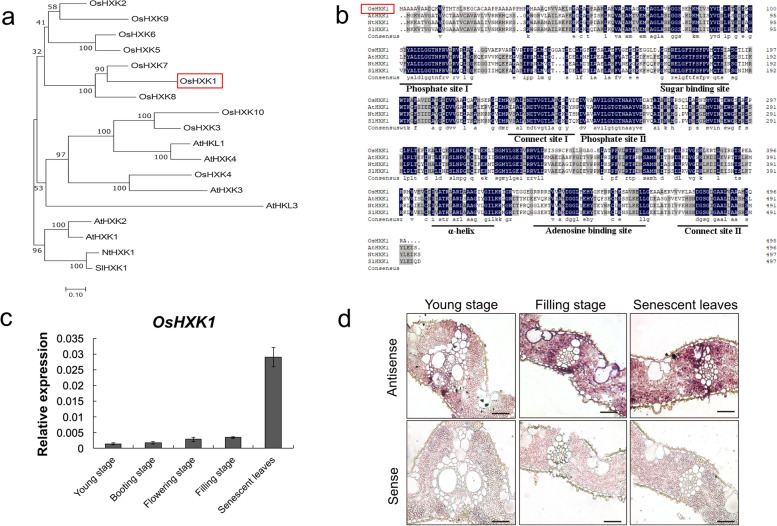
Multiple sequence analysis and expression analysis of OsHXK1. **a** Phylogenetic tree of OsHXK1 and other known hexokinase proteins. OsHXK1 is shown in a red frame. **b** Multiple alignment of OsHXK1 and other known hexokinase protein sequences. The identical amino acids are shaded in black, and similar amino acids are shaded in gray. **c** Relative expression of *OsHXK1* at different leaf stages. All data are presented as means (± SEs) of three independent replicates. **d** In situ analyses of *OsHXK1* in WT leaves at the young and filling stages and in senescent leaves. Bars = 25 μm in **d**

OsHXK1 is one of the ten members of the rice HXK family. To determine the evolutionary relationships among HXK1 in various species, we explored multiple amino acid sequence alignments in the protein domains of 10 rice HXKs and other HXK1 proteins in various species. A phylogenetic analysis showed that OsHXK1 shared the highest homology with OsHXK7 (70.19%) in rice and exhibited slightly higher homology with *Arabidopsis* AtHXK1 (59.18%) than with tobacco NtHXK1 (57.76%) and tomato SlHXK1 (57.97%) (Fig. 1a). The alignment results showed that OsHXK1 possessed two phosphate sites and connected sites (I and II), one sugar-binding site, one adenosine-binding site, and one conserved α-helix site (Fig. 1b). Based on the results from phylogenetic analyses of various species, *Arabidopsis* and rice HXK1 exhibited the highest homology, and HXK1 of *Arabidopsis* was shown to function in regulating leaf senescence [29], which suggested that OsHXK1 might have a similar function in leaf development.

We detected the expression patterns of Os*HXK1* on wild-type leaves at different stages using the qRT-PCR assay; the results indicated that *OsHXK1* expression was extremely low in younger leaves and significantly higher in senescent leaves than at earlier stages (Fig. 1c). In situ hybridization suggested significantly higher expression of *OsHXK1* in senescent leaves, which was consistent with the qRT-PCR results. The *OsHXK1* sense probe, as a negative control, did not observe any detectable signal (Fig. 1d). These results supported the postulation that *OsHXK1* might be involved in rice leaf development.

### Changes in *OsHXK1* expression affect leaf senescence

**Fig. 2:**
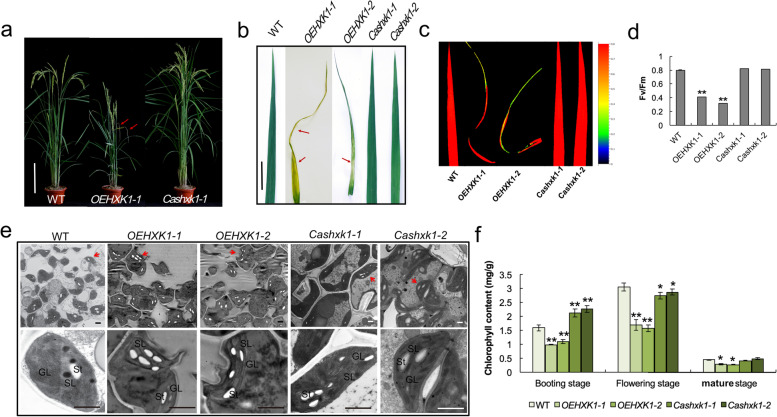
Phenotypic characterization analysis of WT, *OEHXK1–1*, *OEHXK1–2*, *Cashxk1–1*, and *Cashxk1–2.* **a** Plants of the WT, *OEHXK1–1*, and *Cashxk1–1* lines after the flowering stage. **b** Comparison of the flag leaf phenotype of WT, *OEHXK1–1*, *OEHXK1–2*, *Cashxk1–1*, and *Cashxk1–2* plants. **c**-**d**, Chlorophyll fluorescence imaging and Fv/Fm ratio analysis of wild-type, *OEHXK1–1*, *OEHXK1–2*, *Cashxk1–1*, and *Cashxk1–2* plants after the flowering stage. **e** TEM analysis of leaves of WT, *OsHXK1*-overexpressing, and CRISPR/Cas9 plants at the filling stage. GL, grana lamellae, SL, stroma lamellae, St, starch grain. **f** Chlorophyll content analysis of WT, *OsHXK1*-overexpressing, and CRISPR/Cas9 plant leaves from the booting to the mature stage. *, 0.01 < *P* < 0.05. **, *P* < 0.01. The error bars indicate SDs. Bars = 20 cm in **a**, 5 cm in **b**, and 1 μm in **e**

To further investigate the role of *OsHXK1* in leaf senescence, we screened two *OsHXK1*-overexpressing transgenic plants (*OEHXK1–1* and *OEHXK1–2*) and two CRISPR/Cas9 lines (*Cashxk1–1* and *Cashxk1–2*) (Fig. 2a, Additional file 1a). The accumulation of *OsHXK1* transcripts and proteins in these transgenic lines was quantified by qRT-PCR and immunoblotting. The results indicated that the mRNA and protein accumulation of OsHXK1 was increased in the *OsHXK1*-overexpressing plants but decreased in the CRISPR/Cas9 lines (Additional file 1b-d). During rice development, the *OsHXK1*-overexpressing plants displayed precocious leaf senescence symptoms compared with the wild-type plants from the flowering to the filling stage (Fig. 2a-b, Additional file 1 Fig. 1a). The CRISPR/Cas9 lines showed a delayed senescent leaf phenotype (Fig. 2a-b, Additional file 1a). Notably, overexpression of *OsHXK1* also led to severe plant growth retardation, and the leaves appeared yellow with withered leaf tips. Chlorophyll fluorescence imaging could indicate the normality of the chloroplast within the leaf. We also measured the chlorophyll fluorescence parameters in leaves during this period. The results showed that the chlorophyll fluorescence parameters of the photosystem and the Fv/Fm ratio of the *OsHXK1-*overexpressing plants were obviously lower than those of the WT and CRISPR/Cas9 lines (Fig. 2c-d). These results indicated that the chloroplasts in the *OsHXK1-*overexpressing leaves were damaged.

After observing serious premature leaf senescence in the leaves of the *OsHXK1*-overexpressing lines (Fig. 2b), we analyzed the differences in the structure of leaf tissues among the WT, *OsHXK1*-overexpressing, and CRISPR/Cas9 lines by TEM. Magnification of the chloroplast structures showed that the normal granum stacks were intact in WT and *OsHXK1*-CRISPR/Cas9 leaves. In contrast, an increased number of starch bodies, partially defective grana, loosening, and reduced thylakoid stack organization were observed in the *OsHXK1*-overexpressing leaves (Fig. 2e).

A decrease in chlorophyll content is a significant characteristic of leaf senescence [35]. We measured the chlorophyll content of the WT, *OsHXK1*-overexpressing, and *OsHXK1*-CRISPR/Cas9 lines from the booting to the mature stage, and the results revealed a significant decrease in chlorophyll content in *OsHXK1*-overexpressing leaves at the booting, flowering, and mature stages but an increase in the *OsHXK1*-CRISPR/Cas9 lines compared with WT, consistent with the observed phenotypes in leaves (Fig. 2f).

These results indicated that increases in *OsHXK1* expression accelerated premature leaf senescence.

### Acceleration of dark-induced leaf senescence by *OsHXK1*

**Fig. 3:**
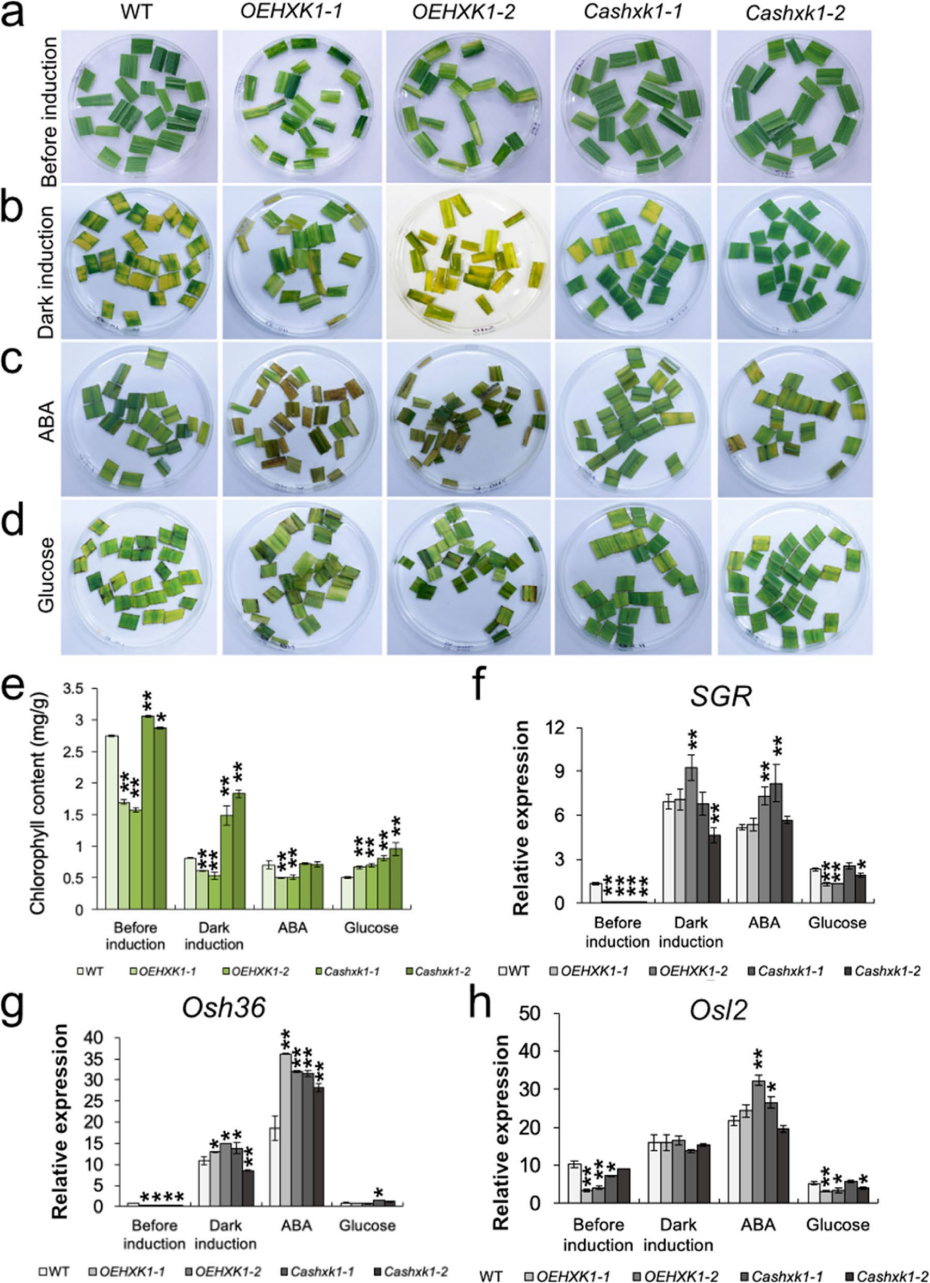
*OsHXK1* participates in dark-induced leaf senescence. **a**-**d** *OsHXK1* promoted dark-induced leaf senescence. Detached flag leaves from WT, *OEHXK1–1*, *OEHXK1–2*, *Cashxk1–1*, and *Cashxk1–2* plants at the flowering stage were incubated with water, 50 μM ABA, and 6% glucose for 5 days in the dark. **e** Chlorophyll content in detached leaves of WT, *OEHXK1–1*, *OEHXK1–2*, *Cashxk1–1*, and *Cashxk1–2* plants after different dark treatments. The values are the means ± SDs of 10 measurements. *, 0.01 < *P* < 0.05. **, *P* < 0.01. *P* values were determined by Student’s *t-*test. The error bars indicate SDs. **f**-**h**, Expression of some *SAG*s (*OsSGR*, *Osh36*, and *Osl2*) in detached leaves of WT and *OsHXK1* mutant plants after different treatments. *, 0.01 < *P* < 0.05. **, *P* < 0.01. The P value was determined by Student’s *t-*test. The error bars indicate SDs

Dark treatment is an efficient method to simulate synchronous senescence in plants and has been applied in numerous studies examining leaf senescence [36–38]. Therefore, we investigated the effects of *OsHXK1* on the progression of dark-induced leaf senescence. The effects of darkness on detached leaves were assayed, and the phenotypes of detached leaves from the WT, *OEHXK1–1*, *OEHXK1–2*, *Cashxk1–1,* and *Cashxk1–2* lines were observed after 5 days of dark treatment. During the dark incubation process, detached leaves from the *OsHXK1*-overexpressing lines became more yellow, and the acceleration rate of leaf senescence in these lines was higher than that in WT plants. Detached leaves of *Cashxk1–1* and *Cashxk1–2* showed a subtle but stay-green phenotype compared with those of WT plants (Fig. 3a and b). Moreover, leaves of the *OsHXK1-*CRISPR/Cas9 lines remained green during exposure to darkness for a longer time, and their chlorophyll content remained high after the treatment compared with those of the WT and *OsHXK1*-overexpressing lines (Fig. 3e). These analyses demonstrated that dark-induced leaf senescence was accelerated in the *OsHXK1*-overexpressing lines and delayed in the *Cashxk1–1* and *Cashxk1–2* lines.

Leaf senescence can be modulated by multiple phytohormones, including ABA and other environmental factors. ABA participates in the process of leaf senescence regulation [9], and hexokinase presents a sensing effect on glucose [29]. We treated the detached leaves with ABA and glucose in the dark (Fig. 3c and d). As an outcome, all leaves exhibited chlorosis and senescence under ABA treatment. The leaves of the *OsHXK1-*CRISPR/Cas9 lines showed an accelerated yellowish coloration, and their chlorophyll content was significantly decreased compared with that of other plant leaves (Fig. 3c and e). Under glucose treatment, the leaves of the WT and *OsHXK1-*overexpressing lines showed delayed yellowing, whereas those of the *OsHXK1-*CRISPR/Cas9 lines exhibited accelerated yellowing. The chlorophyll content of WT and *OsHXK1-*CRISPR/Cas9 leaves under glucose treatment showed a significantly greater decrease versus that in *OsHXK1*-overexpressing leaves compared with the difference before dark induction treatment (Fig. 3d and e). Accordingly, we measured the transcription level of several senescence-associated gene (SAG) markers [39] in WT, *OsHXK1-*overexpressing, and *OsHXK1-*CRISPR/Cas9 leaves after implementing different treatments (Fig. 3f-h, Additional file 2). Although *OsHXK1*-overexpressing leaves exhibited the premature leaf senescence phenotype before the treatment, the expression of *OsSGR*, *Osh36*, and *Osl2* was not significantly increased. The expression of these genes was increased in WT, *OsHXK1*-overexpressing, and CRISPR/Cas9 leaves under the dark and ABA treatments (Fig. 3f-h). After treatment with glucose, the leaves of *OsHXK1*-overexpressing plants showed less sensitivity to glucose (Fig. 3d). The expression patterns of the SAG marker genes in the *OsHXK1*-overexpressing leaves were not the same as those in the CRISPR/Cas9 and WT leaves, and the chlorophyll levels in *OsHXK1*-overexpressing leaves were not seriously decreased compared with those in the CRISPR/Cas9 and WT leaves in the dark (Fig. 3e-h). These results indicated that the senescence pathway mediated by *OsHXK1* was not a conventional pathway and might be related to glucose. Additionally, the abovementioned results indicated that *OsHXK1* had a positive role in leaf senescence.

### *OsHXK1* promotes the accumulation of reactive oxygen species

**Fig. 4:**
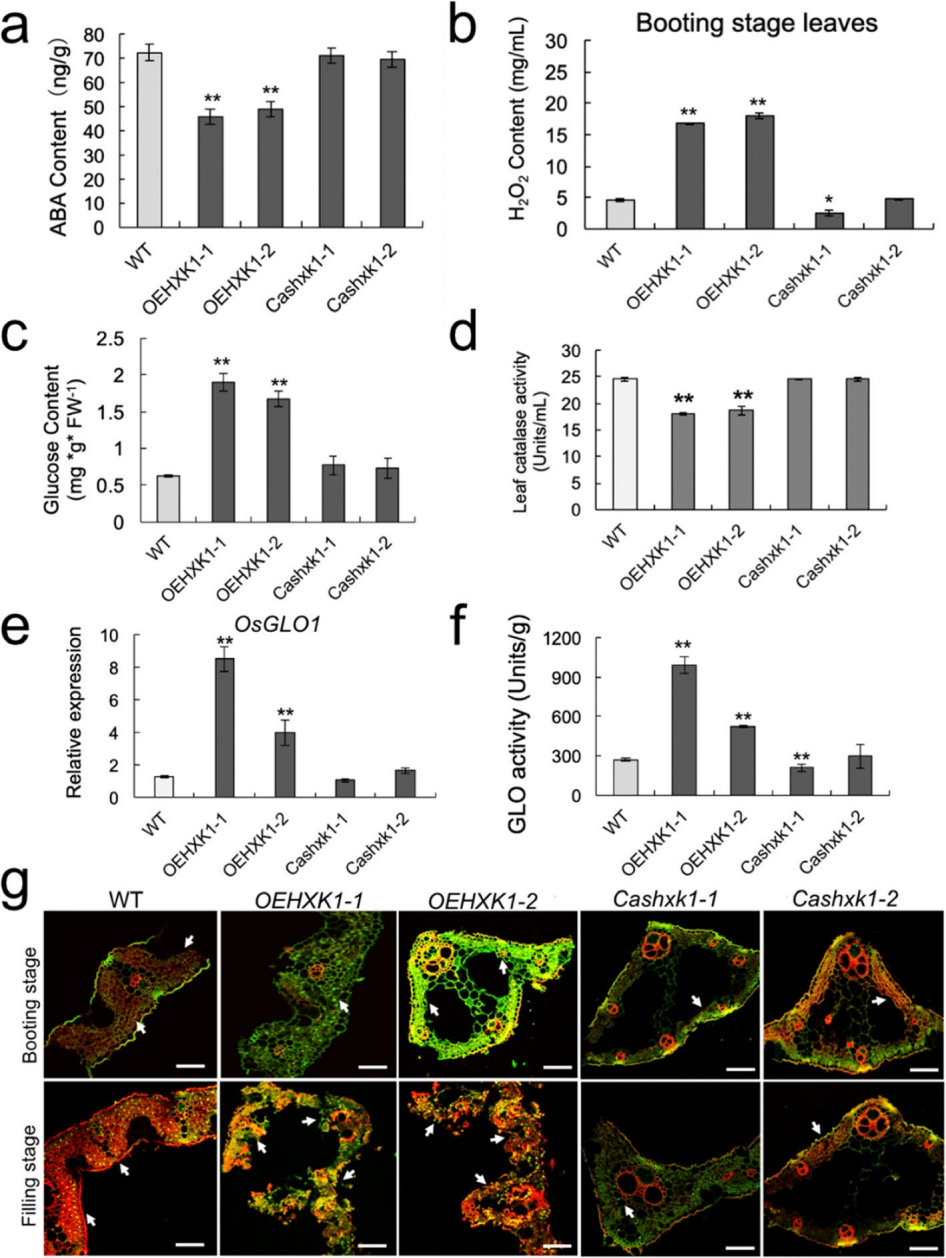
*OsHXK1* increases ROS accumulation to lead to early leaf senescence. **a**-**c**, ABA, glucose, and H_2_O_2_ contents in leaves of WT, *OEHXK1–1*, *OEHXK1–2*, *Cashxk1–1*, and *Cashxk1–2* plants at the booting stage. FW, fresh weight. *, 0.01 < *P* < 0.05, **, *P* < 0.01. The P value was determined by Student’s *t-*test. The error bars indicate SDs. **d** Total extractable leaf catalase activity in leaves of wild-type, *OEHXK1–1*, *OEHXK1–2*, *Cashxk1–1*, and *Cashxk1–2* plants at the filling stage. The data were obtained from three independent replicates. **, *P* < 0.01 according to Student’s *t-*test. The error bars indicate SDs. **e** qRT-PCR analysis of *OsGLO1* in WT, *OEHXK1–1*, *OEHXK1–2*, *Cashxk1–1*, and *Cashxk1–2* leaves at the filling stage. **, *P* < 0.01. The error bars indicate SDs. Each reaction represents three independent replicates. **f** Total extractable leaf GLO activity in leaves of wild-type, *OEHXK1–1*, *OEHXK1–2*, *Cashxk1–1*, and *Cashxk1–2* plants at the filling stage. The data were obtained from three independent replicates. **, *P* < 0.01 according to Student’s *t-*test. The error bars indicate SDs. **g** DNA fragment signals from WT and *OsHXK1* mutant plants at the booting and filling stages. Red fluorescence from the staining of anthers with propidium iodide (PI) was visualized by confocal laser scanning microscopy; the images show the overlays between the green fluorescence from TUNEL staining and PI staining. Bars = 20 μm in **g**

Abscisic acid accumulation could promote leaf senescence [9]. To examine the effect of ABA on leaf senescence, we analyzed the ABA levels in senescent leaves of *OEHXK1–1*, *OEHXK1–2*, *Cashxk1–1*, *Cashxk1–2*, and WT plants. The ABA content in *OEHXK1* plants was obviously decreased compared with that in WT and *OsHXK1-*CRISPR/Cas9 plants (Fig. 4a and Additional file 3a). This finding indicated that ABA was not involved in the regulation of *OsHXK1-*mediated leaf senescence.

Based on these observations, more glucose accelerated ROS production [40], and the ROS level in *OsHXK1*-overexpressing plants was higher at the booting stage (Fig. 4b). Therefore, we quantified the glucose and fructose contents of WT, *OsHXK1-*OE, and *OsHXK1-*CRISPR/Cas9 leaves at the booting stage, and the results revealed a significantly higher glucose content in *OsHXK1-*OE leaves, whereas the fructose content remained unaltered (Fig. 4c, Additional file 3e).

PCD is known to be an important feature of leaf senescence in rice [41]. To determine the effects of altered *OsHXK1* expression in *OsHXK1-*OE plants on the leaf PCD process, we further examined plant DNA fragmentation using a terminal deoxynucleotidyl transferase-mediated dUTP nick end-labeling (TUNEL) assay. Compared with WT, we observed TUNEL-positive signals at an early booting stage in the leaves of both *OEHXK1* plants*.* In contrast, the TUNEL signals for the *OsHXK1-*CRISPR/Cas9 lines were similar to those in WT. The positive PCD signals at the filling stage in *OsHXK1*-OE leaves became more prominent (Fig. 4e).

Optimal ROS levels are required for the PCD process, whereas H_2_O_2_, as a kind of ROS, could be used as a signal for triggering cell death during the aging process. Therefore, we tested the dependency of *OsHXK1*-induced leaf senescence on H_2_O_2_ accumulation. To further explore the involvement of *OsHXK1* in regulating ROS homeostasis, we measured ROS production in *OEHXK1–1*, *OEHXK1–2*, *Cashxk1–1*, *Cashxk1–2*, and WT leaves during the booting and filling stages based on detection of the superoxide anion by NBT and H_2_O_2_ levels by DAB staining. *OEHXK1–1* and *OEHXK1–2* showed more severe stress phenotypes with more lesions and larger lesion areas on the leaf surfaces. Conversely, the *Cashxk1–1* and *Cashxk1–2* leaves remained greener than those of the WT plants. Accordingly, higher ROS levels were detected in *OEHXK1–1* and *OEHXK1–2* leaves than in WT, *Cashxk1–1*, and *Cashxk1–2* leaves (Additional file 3b-c).

We also detected the ROS levels in leaves at different stages. The level of H_2_O_2_ in the leaves of the *OsHXK1*-OE plants at the booting and filling stages was further quantified, and the results showed that the hydrogen peroxide content in the *OsHXK1*-OE plants was higher than that in WT and *OsHXK1-*CRISPR/Cas9 plants (Fig. 4b, Additional file 3d). Furthermore, catalase is a ROS scavenger with activity that is closely related to the accumulation of ROS. Enzyme activity assays showed that the catalase activities of the *OsHXK1-*OE plants were significantly decreased compared with those of the WT plants. In this regard, no difference was observed between WT and *OsHXK1-*CRISPR/Cas9 plants (Fig. 4d). H_2_O_2_, a typical ROS that is biologically toxic to plants, also serves as an important signaling molecule. Its homeostasis is usually regulated by the balance between generation and scavenging rates in the cellular system. GLO and CAT usually act in concert to regulate intracellular H_2_O_2_ levels in plants [42]. We measured the expression level of *OsGLO1* and GLO activity in WT, *OsHXK1*-OE and *OsHXK1-*CRISPR/Cas9 plants, and the results revealed significantly increased expression of *OsGLO1* and GLO activity in *OsHXK1*-OE plants (Fig. 4e, f). These results indicated that the increased ROS levels occurred in *OsHXK1*-OE plants, which might be due to the increased *OsGLO1* expression and GLO activities in these plants.

**Fig. 5:**
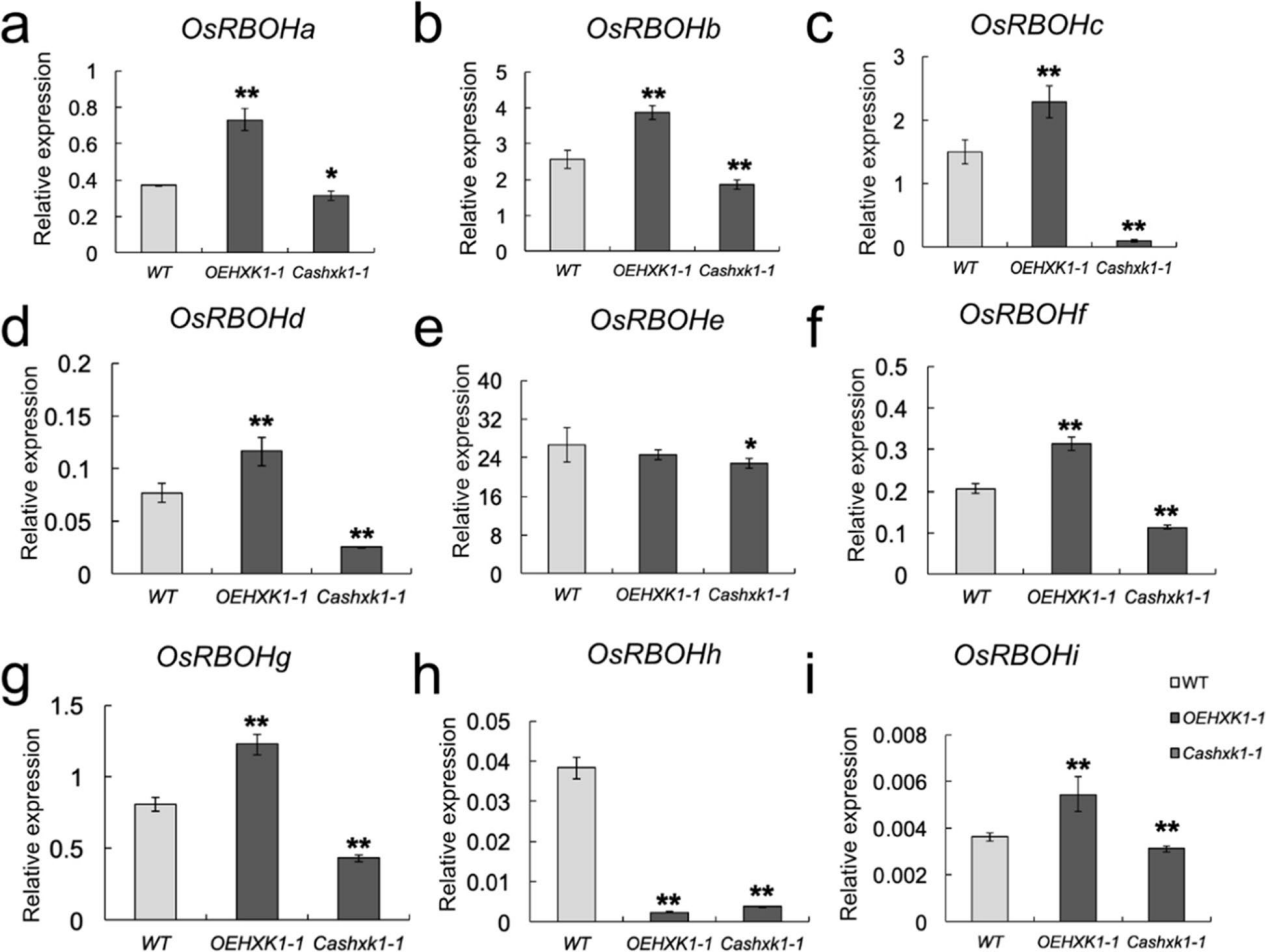
Expression pattern analysis of RBOH genes in WT, *OEHXK1–1*, and *Cashxk1–1* leaves at the booting stage. **a**-**i** qRT-PCR of *OsRBOHa* ~ *OsRBOHi* in WT, *OEHXK1–1*, and *Cashxk1–1* leaves at the booting stage. The error bars indicate SDs. Each reaction represents three independent replicates. Representative data from one of the two biological replicates, which yielded similar results, are shown. *, 0.01 < *P* < 0.05, **, *P* < 0.01. The P value was determined by Student’s *t-*test. The error bars indicate SDs

Plant NADPH oxidase genes, which are known as respiratory burst oxidase homologs (RBOHs), play an important role in ROS production. There are nine RBOH family members in the rice genome: *OsRBOHa*/*OsNOX* to *OsRBOHi*. According to our study, an apoptosis signal was produced in *OsHXK1*-OE leaves at the early booting stage (Fig. 4g). To determine the underlying cause of the increase in ROS levels in *OsHXK1*-OE plants, we performed qRT-PCR analysis to examine the expression levels of *OsRBOH* genes in the leaves at the booting stage. As shown in Fig. 5, seven of the nine OsRBOH genes were significantly increased at the booting stage in *OsHXK1–*overexpressing plants but decreased in the *OsHXK1-*CRISPR/Cas9 lines compared with the WT lines (*OsRBOHa*, *OsRBOHb*, *OsRBOHc*, *OsRBOHd*, *OsRBOHf*, *OsRBOHg*, *OsRBOHi*). The results suggested that changes in *OsRBOH* gene expression patterns may have led to increased ROS accumulation in *OsHXK1*-overexpressing plants.

Collectively, our results showed that overexpression of *OsHXK1* accelerated leaf senescence in an age-dependent manner, and this effect could be associated with an increase in ROS accumulation due to modulation of the expression of OsRBOH genes and *OsGLO1*, and it further regulated the activities of GLO and CAT.

### Catalytically inactive HXK1 mutants exhibit the wild-type phenotype

**Fig. 6:**
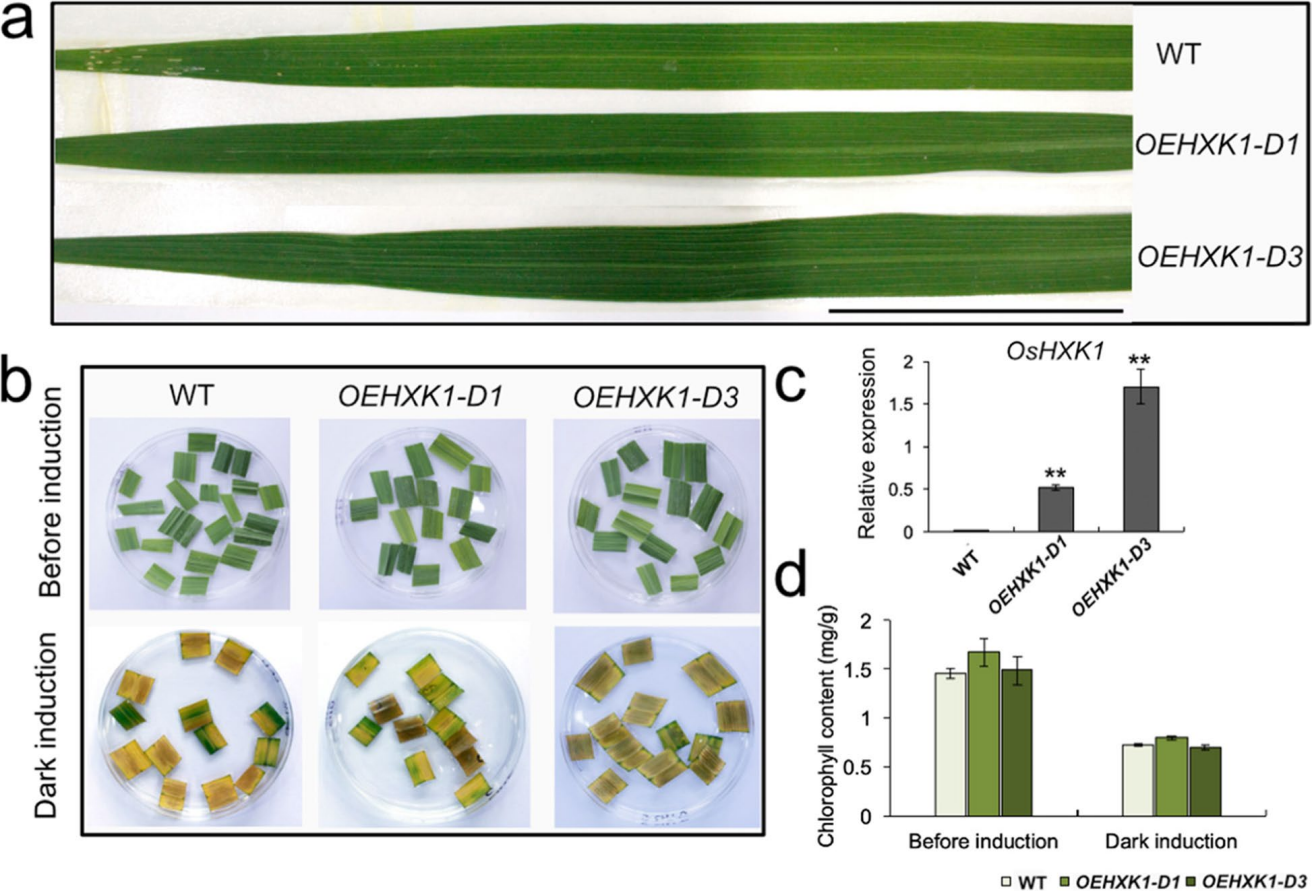
Leaf traits of WT and catalytically inactive *OsHXK1* transgenic lines. **a** Comparison of the leaf phenotypes of WT and catalytically inactive *OsHXK1* transgenic lines (*OsHXK1–D1* and *OsHXK1–D3*). Bars = 5 cm. **b** Detached leaves of WT, *OsHXK1–D1*, and *OsHXK1–D3* plants at the flowering stage were incubated with water before and after 5 days of dark treatment. The data were obtained from three independent replicates. **c** *OsHXK1* expression in WT and catalytically inactive *OsHXK1* transgenic lines. Gene expression levels were measured relative to the rice *OsActin1* transcript level. **d** Chlorophyll contents of detached leaves of WT and catalytically inactive *OsHXK1* transgenic plants before and after dark treatment. The values are the means ± SDs of 10 measurements. *, 0.01 < *P* < 0.05, **, *P* < 0.01. The P value was determined by Student’s *t-*test. The error bars indicate SDs

*Arabidopsis* HXK1 has been shown to possess hexokinase catalytic activity, and Gly^109^ and Ser^182^ are the two key catalytic sites [33]*.* To determine the relationship between the hexokinase catalytic activity of OsHXK1 and plant phenotypic changes, we generated two catalytically inactive overexpression lines (*OEHXK1–D1* and *OEHXK1–D3*). Based on the key catalytic sites in *Arabidopsis*, OsHXK1 was mutated at two active sites in these overexpressing plants, Gly^109^ → Asp^109^ (G109D) and Ser^182^ → Ala^182^ (S182A). Both *OEHXK1–D1* and *OEHXK1–D3* lines displayed increased *OsHXK1* expression levels and normal leaf development, similar to the WT plants (Fig. 6a and c). We also examined the phenotypes of the WT, *OEHXK1–D1*, and *OEHXK1–D3* plants after 5 days of dark treatment. Two catalytically inactive overexpression lines and the WT plants showed the same range of leaf yellowing (Fig. 6b). The chlorophyll content of the WT and two catalytically inactive overexpression lines presented a similar decline in response to dark treatment (Fig. 6d). The abovementioned results suggested that the catalytic activity of *OsHXK1* was a necessary factor for the phenotypic changes in the *OEHXK1* lines and indicated that the functions of HXK1 in rice and *Arabidopsis* were different.

## Discussion

### *OsHXK1* is a key regulator of leaf senescence in rice

Leaf senescence is a highly essential stage and polygenic regulation process that is very closely related to crop yield. If the regulation of leaf senescence becomes uncontrolled, leaves enter senescence at an advanced stage. The phenotypes of early leaf senescence include etiolated leaves, a decreased chlorophyll content, and a damaged chloroplast structure. In addition, dark treatment can accelerate senescence and increase the expression level of senescence-related genes [9, 39, 43, 44]. Our study revealed that *OsHXK1*-overexpressing plants exhibited withered and yellow leaf tips, a decreased chlorophyll content, and a destroyed chloroplast structure compared with wild-type plants (Fig. 2). The evaluated results showed that the phenotypes of the *OsHXK1-*overexpressing leaves were similar to those of prematurely senescent leaves, as described in previous studies, which indicated that *OsHXK1* functioned as a positive regulatory gene of leaf senescence in rice.

The glucose content of the *OsHXK1*-overexpressing leaves increased before the dark treatment (Fig. 4c), and the leaves presented a premature senescence phenotype (Fig. 2b). Interestingly, qRT-PCR analysis showed that the transcript levels of *Osh36*, *OsSGR*, and other senescence-related genes in leaves of the *OsHXK1*-overexpressing plants were not significantly increased before the treatment, which was similar to the expression pattern obtained in the dark combined with glucose treatment. Conversely, the expression of senescence-related genes was significantly upregulated in leaves of the wild-type, *OsHXK1-*overexpressing, and CRISPR/Cas9 plants after the dark and the dark+ABA treatment, and no difference in their expression was detected (Fig. 3f-h and Additional file 2). These results suggested that the senescence process mediated by *OsHXK1* was different from the existing gene regulatory model of senescence, indicating a new model for the control of leaf senescence through regulation of the glucose level.

### *OsHXK1* positively regulates ROS accumulation in rice

Previous studies have shown that ABA is one of the key plant hormones functioning in the leaf senescence process, and the accumulation of ABA can promote the leaf senescence process [45]. The ABA content in the leaves of the *OsHXK1*-overexpressing plants was significantly decreased compared with that in WT leaves (Fig. 4a), which indicated that the leaf senescence process regulated by *OsHXK1* might not be related to the ABA-mediated pathway.

ROS are key signaling molecules in plant cells that also regulate leaf senescence [46]. The increase in ROS accumulation can lead to premature leaf senescence [47, 48]. Our results showed that ROS accumulation in *OsHXK1*-overexpressing plant leaves was significantly increased at the booting stage and led to the earlier production of PCD signals (Fig. 4b and g). The glucose content was also significantly increased at the same stage of leaf development (Fig. 4c). Previous studies have indicated that a high concentration of glucose can induce the accumulation of ROS [40, 49], which implies that the production of ROS in leaves at the booting stage might be due to an increase in glucose content.

NADPH oxidase encoded by the RBOH gene in plants is the main source of ROS production in cells. This enzyme can produce O^2−^ by the oxidation of NADPH, which can lead to a disproportionate formation of H_2_O_2_ and other molecules; thus, this enzyme can participate in the regulation of a variety of biological processes [50, 51]. A study of *Arabidopsis* has shown that *RBOHe*, *RBOHc*, and *RBOHf* can regulate the dynamic levels of ROS and affect the processes of apoptosis and aging [52, 53]. According to the obtained results, seven of the nine genes in the *RBOH* family were upregulated in the leaves of *OsHXK1*-overexpressing plants but downregulated in *OsHXK1-*Cas9 plants (Fig. 5). The RBOH expression changes were similar to ROS accumulation, suggesting that *OsHXK1* could regulate *RBOH* gene expression and thus control ROS production and the leaf senescence process.

It has been reported that approximately 70% of the total H_2_O_2_ is produced by GLO in C3 plants; thus, GLO makes an important contribution to cellular redox status and is involved in multiple H_2_O_2_-related processes in plants [42, 54]. Our results showed that the expression of *OsGLO1* and GLO activity increased significantly in *OsHXK1*-overexpressing plants (Fig. 4e, f). Thus, the increase in ROS levels in premature leaves of *OsHXK1*-OE plants might derive from two mechanisms: the GLO-CAT pathway and RBOH changes.

## Conclusions

Our results revealed the role of *OsHXK1* in rice leaf senescence*.* In conclusion, we developed a hypothetical model for the function of *OsHXK1* in leaf senescence regulation in rice. Overexpression of *OsHXK1* led to the accumulation of glucose and an upregulation of the RBOH gene and *OsGLO1* expression, increasing GLO activity, resulting in excessive accumulation of ROS and triggering earlier PCD. Eventually, the process led to oxidative damage in *OEHXK1–1* and *OEHXK1–2* leaves, which resulted in premature leaf senescence (Additional file 4).

## Methods

### Plant material and growth conditions

The Zhonghua11 (ZH11, *Oryza sativa* ssp. *japonica*) rice plant seeds used in this research as the wild-type and originally cultivated in 1986 were provided by Prof. Yaoguang Liu (South China Agricultural University, China) from the Institute of Crop Sciences, Chinese Academy of Agricultural Sciences. They have been deposited under the code ZH11 (84–213) at the China Rice Data Center (https://www.ricedata.cn/variety/varis/601422.htm). All transgenic lines and wild-type plants were grown in closed field conditions at South China Agricultural University, Guangzhou, China. The transgenic lines included the overexpression lines *OEHXK1–1* and *OEHXK1–2*, the mutant lines *Cashxk1–1* and *Cashxk1–2*, and the overexpression lines with inactive mutations *OEHXK1–D1* and *OEHXK1–D3*; these lines were identified by PCR or qRT-PCR and sequencing by Dr. Shaoyan Zheng (South China Agricultural University, China).

### Characterization of transgenic plant phenotypes

The plants were photographed with a digital camera (Canon 750D). Leaves from different stages were collected based on the plant development morphology. Transmission electronic microscopy (TEM) observations were carried out as described by Zheng et al. [33]. Briefly, leaves from wild-type and mutant plants at the filling stage were cut into approximately 0.2-cm segments and immediately placed in 2 mL of a 4% paraformaldehyde and 2.5% glutaraldehyde fixative. Then, the sample was vacuumed for approximately 20 min and kept at 4 °C overnight. The sample was treated with 1% (w/v) OsO_4_ in phosphate buffer for 1–2 h. After washing 3 times in phosphate buffer (pH 7.2) for 15 min each, the samples were dehydrated using a gradient of ethanol and transferred into a final Eponate 12 resin mixture (#18010, Ted Pell, USA) overnight. The specimens were then placed in capsules with embedding medium and heated at 60 °C overnight. For chloroplast and thylakoid morphology analysis, ultrathin sections were examined and photographed under a Philips FEI Tecnai 12 transmission electron microscope.

### Vector construction and plant transformation

The *OsHXK1* cDNA sequence fragment (Os07g0446800, 1497 bp) amplified from the cDNA library of japonica by KOD polymerase (Toyobo, Japan) was inserted into the pYLox vector between *Hin*dIII and *Mlu*I under the control of the maize *ubiquitin* promoter to construct the *OsHXK1* overexpression vector [33, 55]. To obtain inactive *OsHXK1* mutants, glycine at position 109 (Gly^109^) and serine at position 182 (Ser^182^) were mutated to asparagine (Asp^109^) and alanine (Ala^182^), respectively, through the use of primers with base substitution to induce changes in amino acids by PCR amplification. The PCR products with mutations were cloned into the pYLox vector between *Hin*dIII and *Mlu*I as previously described [33, 55]. To construct the *OsHXK1-*CRISPR/Cas9 vector, two sgRNA sequences targeting *OsHXK1* were cloned into the pYLgRNA*–OsHXK1–*OsU6a and pYLgRNA*–OsHXK1–*OsU3 vectors, and then the two sgRNAs were cloned into the pYLCRISPR/Cas9 pubi-H vector, which has been previously described [33, 56]. The sgRNA and primers used for vector construction are listed in Table S1 in Additional file 5.

All constructs were subjected to Sanger sequencing for confirmation and then transformed into *Agrobacterium tumefaciens EHA105* cells. These cells were then transformed into the wild-type rice variety ZH11 via the *Agrobacterium*-mediated transformation method [57]. Through several generations of hygromycin screening and mRNA expression level detection, we obtained two homozygous transgenic lines for subsequent treatments and analyses. All the primer sequences used in the vector plasmid construction are shown in Table S1 in Additional file 5.

### qRT-PCR assay analysis

Total RNA was obtained from rice leaves (0.1 g) using TRIzol reagent from Invitrogen (USA). Total RNA (500 ng) was treated with DNase I for 2 min, and then the HiScript II Q RT SuperMix kit (Vazyme, # R323–01, Nanjing) was used for reverse transcription. qRT-PCR analyses were then performed using RealStar Green Fast Mixture (GenStar, #A301–101, Beijing) with a qTOWER3G Real-Time PCR Detection System (Analytik Jena, Germany), and the process was repeated three times for each sample. The rice *OsACTIN1* gene (Os10g0510000) was used as the reference gene. Os09g0532000 (*OsSGR*), Os05g0475400, (*Osh36*) Os04g0614600 (*Osl2*), Os01g0227100 (*OsNYC1*), Os06g0354700 (*OsNYC3*), and Os03g0786100 (*OsGLO1*). The OsRBOH genes Os01g0734200 (*a*), Os01g0360200 (*b*), Os05g0528000 (*c*), Os05g0465800 (*d*), Os01g0835500 (*e*), Os08g0453700 (*f*), Os09g0438000 (*g*), Os12g0541300 (*h*), and Os11g0537400 (*i*) were analyzed by qRT-PCR. All experiments in this study were performed with three biological and three technical replicates per biological replicate. The primer sequences used for the qRT-PCR analysis are listed in Table S1 in Additional file 5.

### RNA in situ hybridization assay

The specific RNA probe of *OsHXK1* was amplified using PCR with corresponding specific primers (Table S1) using a Roche DIG RNA Labeling kit (Switzerland) to carry out in vitro transcription. Fresh leaves at different developmental stages (young stage, filling stage, and senescent stage) (cut into approximately 2 × 4 mm) were immediately fixed with 4% paraformaldehyde overnight. After a series of ethanol gradient dehydrations and embedding in Sigma-Aldrich paraffin (USA), the sample was sectioned to a thickness of 5–7 μm. RNA in situ hybridization detection was performed as described previously [33]. The sections on slides were incubated overnight at 45 °C with coverslips in RNA probe hybridization buffer (70–80 μL per slide). Finally, hybridized probe immunological detection was performed using a Roche DIG Nucleic Acid Detection Kit (Switzerland) according to the manufacturer’s protocol.

### Phylogenetic analysis

The phylogenetic tree of HXK1 from multiple eukaryotes was constructed using the maximum likelihood (ML) method. The best-fit models for the evolution of the amino acids were selected by Prot Test server 2.4, which is based on the Akaike Information Criterion (AIC) [58]. The phylogenetic tree of rice and *Arabidopsis* HXK proteins was subsequently constructed based on the model (WAG+I + G + F ≥ JTT + I + G + F–1) [59] in RAxML 8.1.5 with 1000 bootstrap replicates using the ML method. EvolView software (http://www.evolgenius.info/evolview/) was used to construct the phylogenetic trees. The accession numbers related to the phylogenetic analyses were deposited on the National Center for Biotechnology Information website (NCBI, https://www.ncbi.nlm.nih.gov/refseq/about/nonredundantproteins/). The accession numbers for the protein used in the phylogenetic tree construction are listed in Table S2 in Additional file 6.

### Detection of the chlorophyll level and chlorophyll fluorescence imaging

The leaves from wild-type and *OsHXK1* mutant plants were sampled freshly at different periods under field conditions. After removing the main leaf veins, the fresh leaves were cut into small pieces of approximately 2 mm^2^ with a weight of 0.05 g. The leaf pieces from the wild-type and *OsHXK1* mutant plants were then placed in 5.0 mL of 80% acetone, soaked in the dark for 24 h, and shaken every 5 to 12 h until all the leaf photosynthetic pigments were fully dissolved. Two hundred microliters of a sample solution was then used to measure the absorbance (470 nm, 645 nm, and 663 nm) with a visible EPOCH spectrophotometer (BioTek, USA). The chlorophyll amounts were calculated using the following equations: chlorophyll a (Chl a) = (12.7 × A_663_–2.69 × A_645_) × V/W and chlorophyll b (Chl b) = (22.9 × A_645_–4.68 × A_663_) × V/W. The total chlorophyll amount was calculated as the sum of the chlorophyll a and chlorophyll b concentrations. The experiment was performed with two biological lines and included four replicates per group, and Student’s *t*-test was used for the statistical analysis. Chlorophyll fluorescence imaging and Fv/Fm ratio analysis were performed in wild-type, *OEHXK1–1*, *OEHXK1–2*, *Cashxk1–1*, and *Cashxk1–2* plants after the flowering stage. First, the plants to be tested were subjected to dark treatment (at least 30 min). After setting the parameters, leaves of the same size and position were removed from the dark-treated rice plants, and fluorescence imaging photos of each leaf were taken for chlorophyll examination with a chlorophyll fluorescence imager (FluorCam 800 MF, Walz, Germany). The photos were saved, and the corresponding Fv/Fm value was recorded. The error bars represent standard deviations among three replicates.

### Detection of the ABA and glucose contents

Leaves from wild-type plants, *OsHXK1*-overexpressing, and CRISPR/Cas9 plants at different periods were sampled freshly under field conditions. Approximately 100 mg of fresh leaf sample from each line was collected and flash-frozen in liquid nitrogen for the different measurements. ABA extraction was performed according to a previously described protocol [60] and measured using a specific ELISA kit for ABA (Jiangsu Meimian Industrial CO., Ltd., China, MM-0138O1). The ABA levels in the different samples were analyzed using Student’s *t*-test. Three independent biological repeats were performed. To measure the glucose content, approximately 300 mg of fresh leaf sample from each group was flash-frozen in liquid nitrogen. The glucose and fructose contents in leaves were determined at the booting stage as described previously [41]. The glucose and fructose contents in leaves were determined at the booting stage as described previously with slight modifications [61]. Approximately 0.3 g of leaves at the booting stage was ground in liquid nitrogen, and 2 mL of 90% (v/v) ethanol was then added to grind the leaves into a homogenate. The mixture was then transferred to a centrifuge tube (15 mL) and washed twice with 2 mL of 90% ethanol. The sugar-extracting solution was transferred to a water bath at 80 °C for 20 min and centrifuged at 4000 rpm for 10 min, and the supernatant was transferred into a 15-mL tube. Then, the cells were washed with 4 mL 90% ethanol and combined with the supernatant. The supernatant was purified with a RapidVap® rotary evaporation system (Labconco, MO, USA), and 2 mL ultrapure water was added. The mixture was vortexed for 1 min, transferred into a 2-mL tube, and centrifuged (13,000 rpm, 10 min). The supernatant to be tested was filtered through Sep-Pak® 1 cc (100 mg) C18 cartridges. An Agilent Technologies 1200 HPLC system (Waldbronn, Germany) with a four-way pump, refractive index detector (G1362A), column incubator, and automatic sampler was used to detect the sugars. A Transgenomic CARBOSep Coregel 87C cartridge (CHO-99–5860) column was used. Pure water was used as the mobile phase, setting the flow rate (0.6 mL min^− 1^) and column temperature to 80 °C. Pure sucrose and glucose were used as the standard samples to determine the peak time and formulate the standard curve. Four independent biological repeats were included, and all statistical analyses were analyzed using Student’s *t*-test.

### Terminal deoxynucleotidyl transferase-mediated dUTP nick end-labeling analysis

Leaf samples were collected at different developmental stages, fixed with 4% polyformaldehyde, vacuumed for 15 min, and embedded in paraffin. Briefly, fresh leaves at different developmental stages were immediately fixed with 4% paraformaldehyde overnight. After a series of ethanol gradient dehydrations and embedding in Sigma-Aldrich paraffin (USA), the sample was sectioned to a thickness of 5–7 μm. Leaf PCD analysis was performed using a TUNEL assay kit as previously described [33] using the DeadEnd Fluorometric TUNEL system (Promega, #G3250, USA) according to the manufacturer’s instructions. The samples were analyzed under green fluorescence (520 nm) for apoptotic fragments (fluorescein-12-dUTP) in a red (620 nm) background (propidium iodide, PI) using a Zeiss confocal laser-scanning microscope (LSM510, USA). The overlay between the fluorescein and propidium iodide signals was considered a TUNEL-positive signal.

### ROS level measurements

Qualitative analyses of superoxide anion (O^2−^) were performed by nitro blue tetrazolium (NBT) staining, and hydrogen peroxide (H_2_O_2_) was determined by 3,3′-diaminobenzidine (DAB) staining. Briefly, fully expanded leaves collected at different stages were incubated in potassium-citrate buffer (10 mM, pH 6.0) containing NBT (0.5 mM) for NBT staining for 3 h (dark, 25 °C). For DAB staining, approximately 10 cm leaves were detached from different stages and incubated with 0.1% (w/v) DAB (pH 3.8) for 2 h in a growth chamber (light intensity 700 μmol m^− 2^ s^− 1^, temperature 25 °C, and relative humidity 50–60%). Subsequently, the leaves were destained twice with 70% ethanol and photographed. The staining was performed at least four times with two biological replicates. The results were observed under a Leica DNRXA dissecting microscope. A quantitative assay for H_2_O_2_ formation was performed using a hydrogen peroxide assay kit (#S0038, Beyotime Biotech, Shanghai, China) according to the manufacturer’s instructions. Briefly, approximately 0.1 g of leaves was collected at different stages and ground in liquid nitrogen. Then, the powder was extracted from 1 mL of extraction buffer from the kit. After centrifugation (12,000 g, 15 min, 4 °C), the supernatant was used to determine H_2_O_2_ levels by the spectrophotometer method. The CAT activity assay was detected using a UV spectrophotometer in a reaction mixture containing 50 mM PBS (pH 7.4) and 25 mM H_2_O_2_ at 30 °C. Consumption of H_2_O_2_ was measured at 240 nm as previously described [42], and the GLO activity assay was performed according to a pervious report [54]. Briefly, 0.1 g of leaves were detached from the fully expanded leaves (flowering stage) and homogenized in 1 mL of 50 mM PBS (pH 7.4) at 4 °C. The homogenates were centrifuged (12,000 g, 20 min, at 4 °C), and the supernatants were used as enzyme extracts. The reaction mixture contained 50 mM PBS (pH 7.8), 1 mM 4-amino-antipyrine, 0.1 mM FMN, 2 mM phenol, 5 units of horseradish peroxidase, and 5 mM glycolate per 1 mL. The reaction was started by adding enzyme and measured at 520 nm at intervals of 5 s for 1 min. All measurements were conducted at least four times and analyzed with Student’s *t*-test.

### Dark-induced leaf senescence analysis

The fully expanded flag leaves were carefully removed from each plant. Then, the detached flag leaves were cut into ∼2-cm^2^ pieces, and the leaves were cultured on 25 mL of ddH_2_O, 50 μM ABA (Abscisic acid, #90769, Sigma-Aldrich Trading Co. Ltd. Shanghai), or 6% glucose solution with the adaxial side up in Petri dishes. All samples were incubated in the dark at 28 °C for 5 days. The changes in leaf color and state of each plant were observed by the naked eye every day. Each 10 pieces of leaves were harvested for chlorophyll level detection and qRT-PCR analysis. All measurements were performed at least three times and analyzed with Student’s *t*-test.

## Supplementary Information

**Additional file 1 **Phenotypic comparison among WT, *OsHXK1*-OE, and *OsHXK1*-CRISPR/Cas9 plants.

**Additional file 2 **Expression analysis of some *SAGs* in detached leaves of the and *OsHXK1* mutant plants after different treatments.

**Additional file 3 **Analyses of ROS, ABA, and fructose in WT, *OEHXK1–1*, *OEHXK1–2*, *Cashxk1–1*, and *Cashxk1–2* plants.

**Additional file 4 **Proposed model of the regulation of leaf senescence by *OsHXK1* in rice.

**Additional file 5: Table S1.** Primers used in this study.

**Additional file 6: Table S2.** The protein accession numbers used in the phylogenetic analysis.

## Data Availability

All data supporting the conclusions of this article are included within the article and the additional files. The sequence data used in this study can be found in the Rice Annotation Project (https://rapdb.dna.affrc.go.jp/viewer/gbrowse) and have been deposited in the GenBank database. SAG marker gene accession numbers: Os09g0532000 (*OsSGR*), Os05g0475400, (*Osh36*) Os04g0614600 (*Osl2*), Os01g0227100 (*OsNYC1*), Os06g0354700 (*OsNYC3*), and Os03g0786100 (*OsGLO1*). The OsRBOH genes (*OsRBOHa* to *i*): Os01g0734200 (*a*), Os01g0360200 (*b*), Os05g0528000 (*c*), Os05g0465800 (*d*), Os01g0835500 (*e*), Os08g0453700 (*f*), Os09g0438000 (*g*), Os12g0541300 (*h*), and Os11g0537400 (*i*) are available in the Rice Annotation Project (RAP) repository (https:// rapdb. Dna. affrc. go. jp/ index. html).

## Abbreviations

HXK: Hexokinase
TEM: Transmission electron microscope
PCD: Programmed cell death
PI: Propidium iodide
ABA: Abscisic acid
TUNEL: Terminal deoxynucleotidyl transferase-mediated dUTP nick end-labeling
ROS: Reactive oxygen species
NBT: Nitro blue tetrazolium
DAB: 3,3′*–*diaminobenzidine
RBOH: Respiratory burst oxidase homolog
ML: Maximum likelihood

## References

[1] Wingler A, Purdy S, MacLean JA, Pourtau N (2006). The role of sugars in integrating environmental signals during the regulation of leaf senescence. J Exp Bot.

[2] Mae T (1997). Physiological nitrogen efficiency in rice: nitrogen utilization, photosynthesis and yield potential. Plant Soil.

[3] Lee S, Masclaux-Daubresse C (2021). Current understanding of leaf senescence in rice. Int J Mol Sci.

[4] Nooden LD, Guiamet JJ, John I (1997). Senescence mechanisms. Physiol Plantarum.

[5] Lohman KN, Gan S, John MC, Amasino RM (1994). Molecularanalysis of natural leaf senescence in *Arabidopsis thaliana*. Plant Physiol.

[6] Gan S, Amasino RM (1995). Inhibition of leaf senescence by autoregulated production of cytokinin. Science..

[7] Hinderhofer K, Zentgraf U (2001). Identi-cation of a transcription factor specically expressed at the onset of leaf senescence. Planta..

[8] Guo Y, Gan S (2006). AtNAP, a NAC family transcription factor, has an importantvrolevin leaf senescence. Plant J.

[9] Liang C, Wang Y, Zhu Y, Tang J, Hu B, Liu L (2014). OsNAP connects abscisic acid and leaf senescence by fine-tuning abscisic acid biosynthesis and directly targeting senescence-associated genes in rice. Proc Natl Acad Sci U S A.

[10] Shimoda Y, Ito H, Tanaka A (2016). Arabidopsis *STAY-GREEN*, Mendel’s green cotyledon gene, encodes magnesium-dechelatase. Plant Cell.

[11] Ansari MI, Lee R, Chen SG (2005). A novel senescence-associated gene encoding γ-aminobutyric acid (gaba):pyruvate transaminase is upregulated during rice leaf senescence. Physiol Plantarum.

[12] Lee RH, Lin MC, Chen SCG (2004). A novel alkaline α-galactosidase gene is involved in rice leaf senescence. Plant Mol Biol.

[13] Kong Z, Li M, Yang W, Xu W, Xue Y (2006). A novel nuclear-localized CCCH-type zinc finger protein, OsDOS, is involved in delaying leaf senescence in rice. Plant Physiol.

[14] Kusaba M, Ito H, Morita R, Lida S, Sato Y, Fujimoto M (2007). Rice non-yellow coloring1 is involved in light-harvesting complex ii and grana degradation during leaf senescence. Plant Cell.

[15] Morita R, Sato Y, Masuda Y, Nishimura M, Kusaba M (2009). Defect in non-yellow coloring 3, an α/β hydrolase-fold family protein, causes a stay-green phenotype during leaf senescence in rice. Plant J.

[16] Sato Y, Morita R, Katsuma S, Nishimura M, Tanaka A, Kusaba M (2009). Two shortchain dehydrogenase/reductases, NON-YELLOW COLORING 1 and NYC1-LIKE, are required for chlorophyll b and light-harvesting complex II degradation during senescence in rice. Plant J.

[17] Contento AL, Kim SJ, Bassham DC (2004). Transcriptome profiling of the response of Arabidopsis suspension culture cells to Suc starvation. Plant Physiol.

[18] Wingler A, Marès M, Pourtau N (2004). Spatial patterns and metabolic regulation of photosynthetic parameters during leaf senescence. New Phytol.

[19] Pourtau N, Marès M, Purdy S, Quentin N, Ruël A, Wingler A (2004). Interactions of abscisic acid and sugar signalling in the regulation of leaf senescence. Planta..

[20] Quirino BF, Reiter WD, Amasino RM (2001). One of two tandem Arabidopsis genes homologous to monosaccharide transporters is senescence-associated. Plant Mol Biol.

[21] Stessman D, Miller A, Spalding M, Rodermel S (2002). Regulation of photosynthesis during Arabidopsis leaf development in continuous light. Photosynth Res.

[22] Jang J-C, Sheen J (1994). Sugar sensing in higher plants. Plant Cell.

[23] Sun YJ, Hord CL, Chen CB, Ma H (2007). Regulation of *Arabidopsis* early anther development by putative cell-cell signaling molecules and transcriptional regulators. J Integr Plant Biol.

[24] Rolland F, Moore B, Sheen J (2002). Sugar sensing and signaling in plants. Plant Cell.

[25] Halford NG, Purcell PC, Hardie DG (1999). Is hexokinase really a sugar sensor in plants?. Trends Plant Sci.

[26] Smeekens S, Ma J, Hanson J, Rolland F (2010). Sugar signals and molecular networks controlling plant growth. Curr Opin Plant Biol.

[27] Frommer WB, Schulze WX, Lalonde S (2003). Hexokinase, Jack of all trades. Science..

[28] Cho JI, Ryoo N, Ko S, Lee S-K, Lee J, Jung K-H (2006). Structure, expression, and functional analysis of the hexokinase gene family in rice (*Oryza sativa* L.). Planta..

[29] Moore B, Zhou L, Rolland F, Hall Q, Cheng W-H, Liu Y-X (2003). Role of the Arabidopsis glucose sensor HXK1 in nutrient, light, and hormonal signaling. Science..

[30] Damariweissler H, Ginzburg A, Gidoni D, Mett A, Krassovskaya I, Weber APM (2007). Spinach SoHXK1 is a mitochondria-associated hexokinase. Planta..

[31] Cho YH, Yoo SD, Sheen J (2006). Regulatory functions of nuclear hexokinase1 complex in glucose signaling. Cell..

[32] Xu FQ, Li XR, Ruan YL (2008). RNAi-mediated suppression of hexokinase gene OsHXK10 in rice leads to non-dehiscent anther and reduction of pollen germination. Plant Sci.

[33] Zheng S, Li J, Ma L, Wang H, Zhou H, Ni E (2019). OsAGO2 controls ROS production and the initiation of tapetal PCD by epigenetically regulating *OsHXK1* expression in rice anthers. Proc Natl Acad Sci U S A.

[34] Zheng S, Ye C, Lu J, Liufu J, Lin L, Dong Z (2021). Improving the rice photosynthetic efficiency and yield by editing *OsHXK1* via CRISPR/Cas9 system. Int J Mol Sci.

[35] Yang S, Fang G, Zhang A, Ruan B, Jiang H, Ding S (2020). Rice EARLY SENESCENCE 2, encoding an inositol polyphosphate kinase, is involved in leaf senescence. BMC Plant Biol.

[36] Biswal UC, Mohanty P (1976). Dark stress-induced senescence of detached barley leaves. II. Alteration in absorption characteristic and photochemical activity of chloroplasts isolated from senescing leaves. Plant Sci Lett.

[37] Ma X, Balazadeh S, Mueller-Roeber B (2019). Tomato fruit ripening factor NOR controls leaf senescence. J Exp Bot.

[38] Weaver LM, Amasino RM (2001). Senescence is induced in individually darkened Arabidopsis leaves, but inhibited in whole darkened plants. Plant Physiol.

[39] Guo Y, Gan S (2005). Leaf senescence: signals, execution, and regulation. Curr Top Dev Biol.

[40] Huang L, Yu LJ, Zhang X, Fan B, Wang FZ, Dai YS (2019). Autophagy regulates glucose-mediated root meristem activity by modulating ros production in *Arabidopsis*. Autophagy..

[41] Zhang Y, Wang HL, Li Z, Guo H (2020). Genetic network between leaf senescence and plant immunity: crucial regulatory nodes and new insights. Plants..

[42] Zhang Z, Xu Y, Xie Z, Li X, He Z, Peng X (2016). Association-dissociation of glycolate oxidase with catalase in rice: a potential switch to modulate intracellular H_2_O_2_ levels. Mol Plant.

[43] Hong Y, Zhang Y, Sinumporn S, Yu N, Zhan X, Shen X (2018). Premature leaf senescence 3, encoding a methyltransferase, is required for melatonin biosynthesis in rice. Plant J.

[44] Mao C, Lu S, Lv B, Zhang B, Shen J, He J (2017). A rice nac transcription factor promotes leaf senescence via ABA biosynthesis. Plant Physiol.

[45] Lim PO, Kim HJ, Nam HG (2007). Leaf senescence. Annu Rev Plant Biol.

[46] Wang Y, Lin A, Loake GJ, Chu C (2013). H_2_O_2−_induced leaf cell death and the crosstalk of reactive nitric/oxygen species. J Inter Plant Biol.

[47] Leng Y, Yang Y, Ren D, Huang L, Dai L, Wang Y (2017). A rice pectate lyase-like gene is required for plant growth and leaf senescence. Plant Physiol.

[48] Zhou Q, Yu Q, Wang Z, Pan Y, Lv W, Zhu L (2013). Knockdown of GDCH gene reveals reactive oxygen species-induced leaf senescence in rice. Plant Cell Environ.

[49] Couée I, Sulmon C, Gouesbet G, El Amrani A (2006). Involvement of soluble sugars in reactive oxygen species balance and responses to oxidative stress in plants. J Exp Bot.

[50] Hampton MB, Kettle AJ, Winterbourn CC (1998). Inside the neutrophil phagosome: oxidants, myeloperoxidase, and bacterial killing. Blood..

[51] Suzuki N, Miller G, Morales J, Shulaev V, Torres MA, Mittler R (2011). Respiratory burst oxidases: the engines of ROS signaling. Curr Opin Plant Biol.

[52] Niu F, Cui X, Zhao P, Sun M, Yang B, Deyholos MK (2020). WRKY42 transcription factor positively regulates leaf senescence through modulating SA and ROS synthesis in *Arabidopsis thaliana*. Plant J.

[53] Xie HT, Wan ZY, Li S, Zhang Y (2014). Spatiotemporal production of reactive oxygen species by NADPH oxidase is critical for tapetal programmed cell death and pollen development in Arabidopsis. Plant Cell.

[54] Zhang Z, Li X, Cui L, Meng S, Ye N, Peng X (2017). Catalytic and functional aspects of different isozymes of glycolate oxidase in rice. BMC Plant Biol.

[55] Yu L, Jiang J, Zhang C, Jiang L, Ye N, Lu Y (2010). Glyoxylate rather than ascorbate is an efficient precursor for oxalate biosynthesis in rice. J Exp Bot.

[56] Ma X, Zhang Q, Zhu Q, Liu W, Chen Y, Qiu R (2015). A robust CRISPR/Cas9 system for convenient, high-efficiency multiplex genome editing in monocot and dicot plants. Mol Plant.

[57] Li J, Jiang D, Zhou H, Li F, Yang J, Hong L (2011). Expression of RNA-interference/antisense transgenes by the cognate promoters of target genes is a better gene-silencing strategy to study gene functions in rice. PLoS One.

[58] Darriba D, Taboada GL, Doallo R, Posada D (2011). ProtTest 3: fast selection of best-fit models of protein evolution. Bioinformatics..

[59] Stamatakis A (2006). RAxML-VI-HPC: maximum likelihood-based phylogenetic analyses with thousands of taxa and mixed models. Bioinformatics..

[60] Wu J, Hettenhausen C, Meldau S, Baldwin IT (2007). Herbivory rapidly activates MAPK signaling in attacked and unattacked leaf regions but not between leaves of *Nicotiana attenuata*. Plant Cell.

[61] Yang Z, Wang T, Wang H, Huang X, Qin Y, Hu G (2013). Patterns of enzyme activities and gene expressions in sucrose metabolism in relation to sugar accumulation and composition in the aril of *Litchi chinensis* Sonn. J Plant Physiol.

