# Supplementary material for: Hexokinase gene OsHXK1 positively regulates leaf senescence in rice
Source: BMC Plant Biol. 2021 Dec 8;21:580. doi: 10.1186/s12870-021-03343-5 (PMC8653616; doi:10.1186/s12870-021-03343-5)
Supplement: Supplementary file 1 — Additional file 1 Phenotypic comparison among WT, OsHXK1-OE, and OsHXK1-CRISPR/Cas9 plants. [file 12870_2021_3343_MOESM1_ESM.zip › Additional file 1.docx]

**
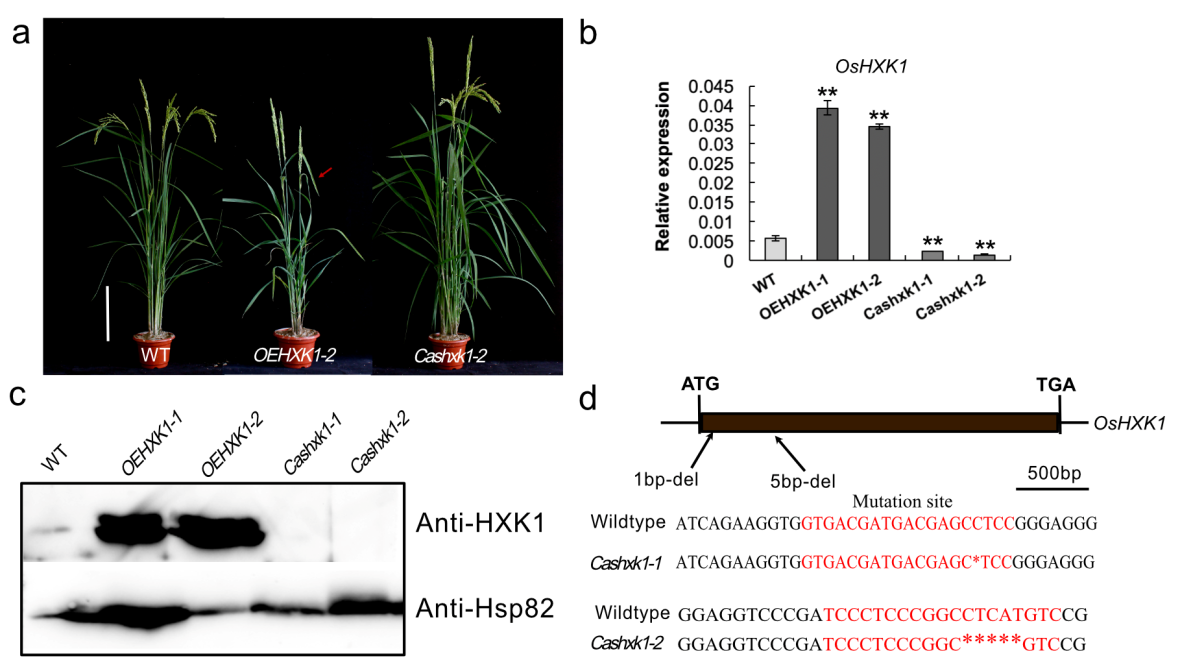
**

**Additional file 1.** **Phenotypic comparison among wild-type (WT), *OsHXK1*-OE, and *OsHXK1*-CRISPR/Cas9 plants.**

a, Plants of the WT, *OEHXK1–2*, and *Cashxk1–2* lines after the flowering stage. b, qRT-PCR analysis of *OsHXK1* transcript levels in the WT, *OEHXK1–1*, *OEHXK1–2*, *Cashxk1–1*, and *Cashxk1–2* lines. *OsActin1* served as a control. Error bars represent standard deviations among replicates (n = 3) *, 0.01 < P < 0.05. **, P < 0.01. The P values were determined by Student’s *t-*test. Error bars indicate SDs. c, Western blot analysis of the WT, *OEHXK1–1*, *OEHXK1–2*, *Cashxk1–1*, and *Cashxk1–2* plants showed the accumulation of OsHXK1 proteins at the filling stage. Anti-Hsp82 was used as the loading control. d, Mutational sites in *OsHXK1*-CRISPR/Cas9 transgenic plants. The WT sequence is shown at the top with the PAM sequence, and the target sequence is highlighted in red. The deletions of 1 bp in *Cashxk1–1* and 5 bp in *Cashxk1–2* are highlighted in red. The “*” shows the deleted bases. Bars = 20 cm in a.
