# Supplementary figures and images for: Hexokinase gene OsHXK1 positively regulates leaf senescence in rice
Source: BMC Plant Biol. 2021 Dec 8;21:580. doi: 10.1186/s12870-021-03343-5 (PMC8653616; doi:10.1186/s12870-021-03343-5)

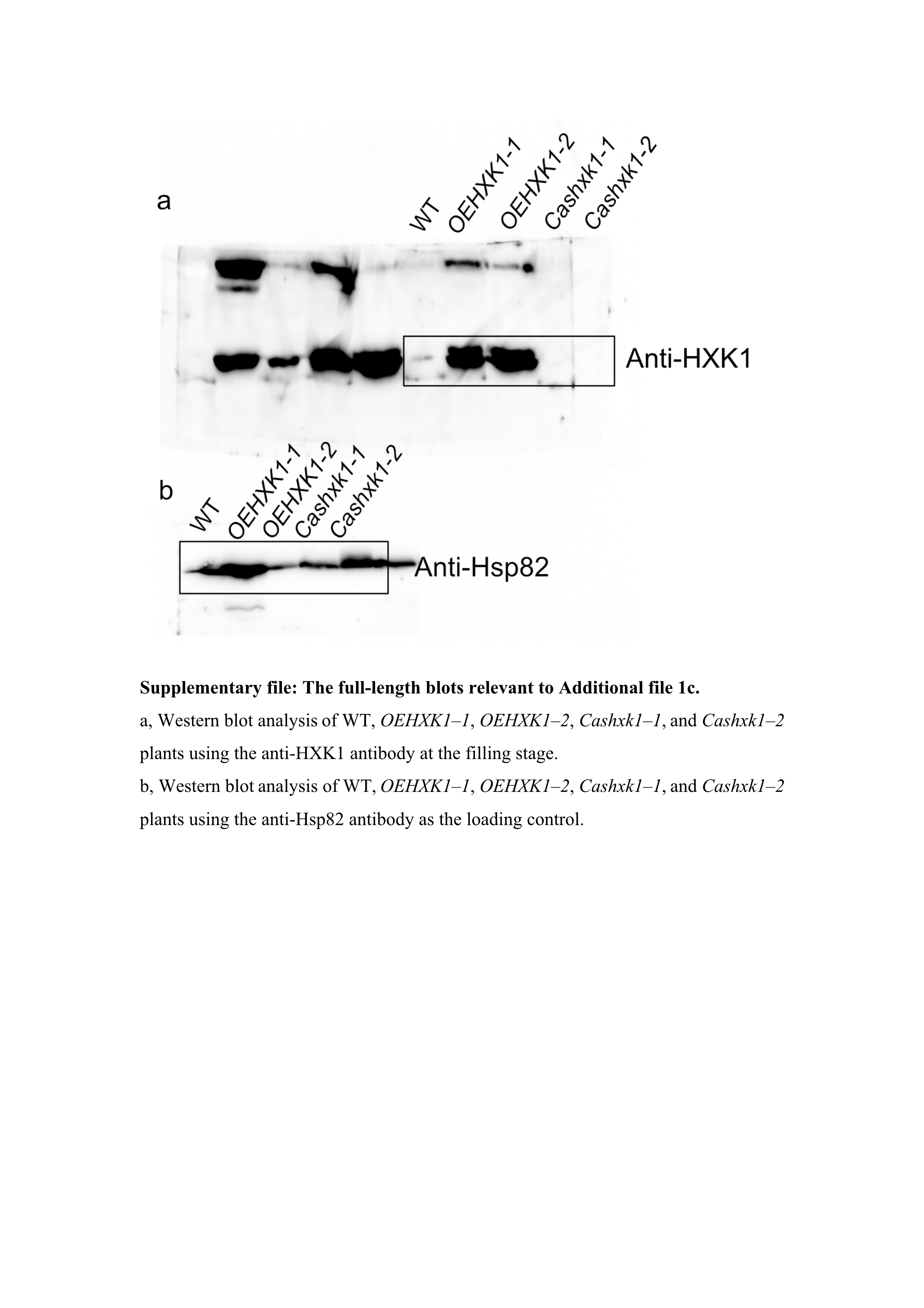

Supplement: Supplementary file 1 — Additional file 1 Phenotypic comparison among WT, OsHXK1-OE, and OsHXK1-CRISPR/Cas9 plants. [file 12870_2021_3343_MOESM1_ESM.zip › The full-length blots relevant to Additional file 1c.tiff]

**
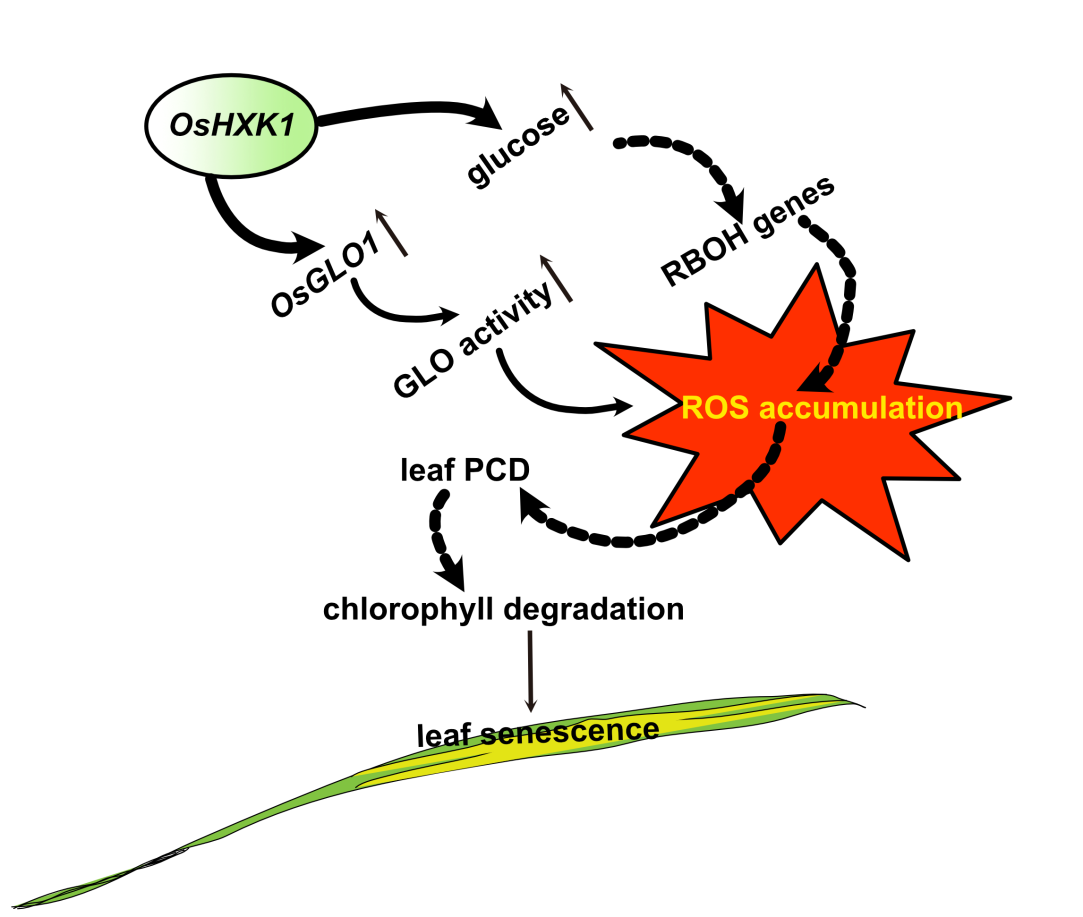
**

**Additional file 4. Proposed model of the regulation of leaf senescence by *OsHXK1* in rice.**

Supplement: Supplementary file 4 — Additional file 4 Proposed model of the regulation of leaf senescence by OsHXK1 in rice. [file 12870_2021_3343_MOESM4_ESM.docx]
