# Supplementary material for: Hexokinase gene OsHXK1 positively regulates leaf senescence in rice
Source: BMC Plant Biol. 2021 Dec 8;21:580. doi: 10.1186/s12870-021-03343-5 (PMC8653616; doi:10.1186/s12870-021-03343-5)
Supplement: Supplementary file 2 — Additional file 2 Expression analysis of some SAGs in detached leaves of the and OsHXK1 mutant plants after different treatments. [file 12870_2021_3343_MOESM2_ESM.docx]

**
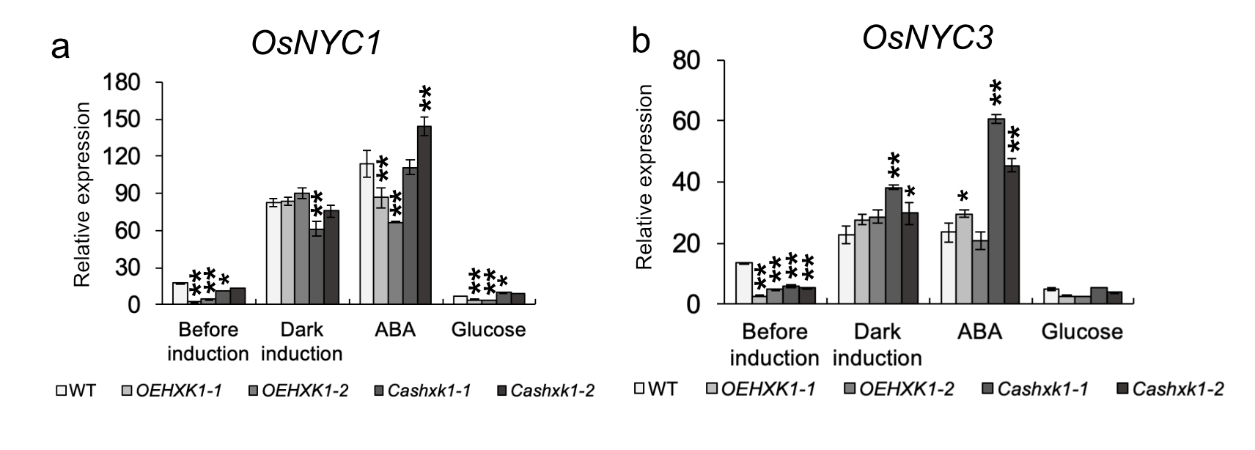
**

**Additional file 2. Expression analysis of some *SAGs* in detached leaves of WT and *OsHXK1* mutant plants after different treatments.**

a-b, Expression of some *SAGs* (*OsNYC1* and *OsNYC3*) in detached leaves of WT, *OEHXK1–1*, *OEHXK1–2*, *Cashxk1–1*, and *Cashxk1–2* plants after different treatments. *, 0.01 < P < 0.05. **, P < 0.01. The P value was determined by Student’s *t-*test. Error bars represent standard deviations among replicates (n = 3).
