# Supplementary material for: Hexokinase gene OsHXK1 positively regulates leaf senescence in rice
Source: BMC Plant Biol. 2021 Dec 8;21:580. doi: 10.1186/s12870-021-03343-5 (PMC8653616; doi:10.1186/s12870-021-03343-5)
Supplement: Supplementary file 3 — Additional file 3 Analyses of ROS, ABA, and fructose in WT, OEHXK1–1, OEHXK1–2, Cashxk1–1, and Cashxk1–2 plants. [file 12870_2021_3343_MOESM3_ESM.docx]

**
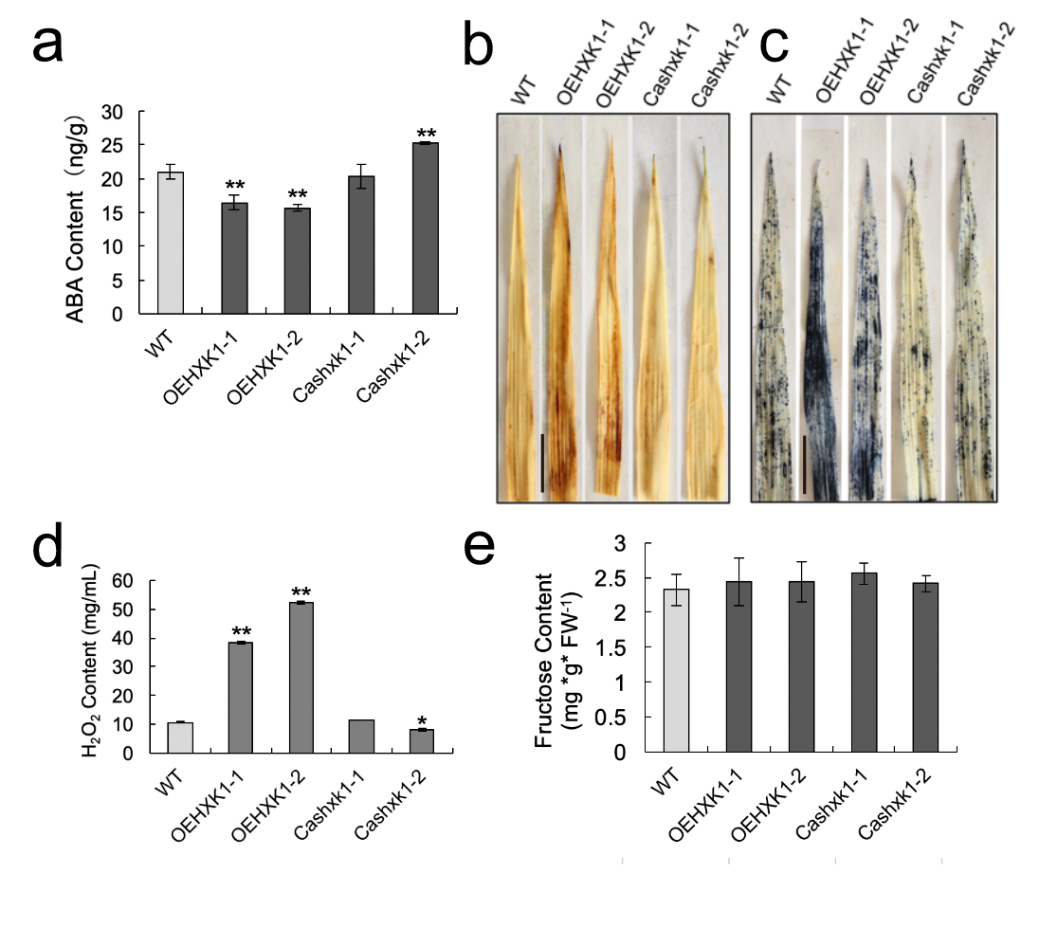
**

**Additional file 3. Analyses of ROS, ABA, and fructose in WT, *OEHXK1–1*, *OEHXK1–2*, *Cashxk1–1*, and *Cashxk1–2* plants.**

a, ABA content in WT, *OEHXK1–1*, *OEHXK1–2*, *Cashxk1–1*, and *Cashxk1–2* leaves at the filling stage. *, 0.01 < P < 0.05, **, P < 0.01. The P value was determined by Student’s *t-*test. b-c, DAB and NBT staining of WT, *OEHXK1–1*, *OEHXK1–2*, *Cashxk1–1*, and *Cashxk1–2* leaves indicating ROS levels. Bars = 3 cm. d, H_2_O_2_ content in WT, *OEHXK1–1*, *OEHXK1–2*, *Cashxk1–1*, and *Cashxk1–2* leaves at the filling stage. *, 0.01 < P < 0.05, **, P < 0.01. The P value was determined by Student’s *t-*test. Error bars indicate SDs. e, Fructose content in WT, *OEHXK1–1*, *OEHXK1–2*, *Cashxk1–1*, and *Cashxk1–2* leaves at the booting stage. FW, fresh weight. *, 0.01 < P < 0.05, **, P < 0.01. The P value was determined by Student’s *t-*test. Error bars indicate SDs.
