# Supplementary material for: Hexokinase gene OsHXK1 positively regulates leaf senescence in rice
Source: BMC Plant Biol. 2021 Dec 8;21:580. doi: 10.1186/s12870-021-03343-5 (PMC8653616; doi:10.1186/s12870-021-03343-5)
Supplement: Supplementary file 5 — Additional file 5: Table S1. Primers used in this study. [file 12870_2021_3343_MOESM5_ESM.docx]

**Additional file 5. Table S1. Primers used in this study**

| Primer name | Sequence (5'-3') | Objective |
| --- | --- | --- |
| HXH1cas9-U3F | GTTGGTGACGATGACGAGCCTCC | Vector construction |
| HXH1cas9-U3R | AAACGGAGGCTCGTCATCGTCAC | Vector construction |
| HXH1cas9-U6bF | GGCATCCCTCCCGGCCTCATGTC | Vector construction |
| HXH1cas9-U6bR | AAACGACATGAGGCCGGGAGGGA | Vector construction |
| OEHXK1F | CTTCGTGCGGTTCAAAGCTTGTTCG | Vector construction |
| OEHXK1R | AAAAACGCGTTTATGCTCTCTGCGAGTGAG | Vector Construction |
| HXKG109D-F | CTCGACCTCGGCGACACCAACTTCC | Vector Construction |
| HXKG109D-R | GGAAGTTGGTGTCGCCGAGGTCGAG | Vector Construction |
| HXKS182A-F | GTTCACCTTCGCCTTCCCCGTGCACCAAAC | Vector Construction |
| HXKS182A-R | GTTTGGTGCACGGGGAAGGCGAAGGTGAAC | Vector Construction |
| qHXK1F | ATCGCCAAGCTACACCCATC | qRT-PCR |
| qHXK1R | GGTTGAGGCTCTCCTGATCG | qRT-PCR |
| insu HXK1T7F | AATTAATACGACTCACTATAGTCGAGCACTACGGCAAGTTC | *In situ* hybridization |
| insu HXK1R | ACACACGCATATTTCACCAC | *In situ* hybridization |
| insu HXK1F | TCGAGCACTACGGCAAGTTC | *In situ* hybridization |
| insu HXK1T7R | AATTAATACGACTCACTATAGACACACGCATATTTCACCAC | *In situ* hybridization |
| ActinF | CACATTCCAGCAGATGTGGA | qRT-PCR |
| ActinR | GCGATAACAGCTCCTCTTGG | qRT-PCR |
| RBOHaF | ATCCGCAAAATAAGCACCTCT | qRT-PCR |
| RBOHaR | CAGTAGCCCATCACATCAAAGA | qRT-PCR |
| RBOHbF | GGCTTCAATGCCTTCTGGT | qRT-PCR |
| RBOHbR | ATGGCTCCTAAACAACCGA | qRT-PCR |
| RBOHcF | CCAGTGGGTGGGAAAAGTG | qRT-PCR |
| RBOHcR | GTCCGATTGGCGGGTAAA | qRT-PCR |
| RBOHdF | CACAAGGTTATCGCACTGAC | qRT-PCR |
| RBOHdR | AGCGATGAGTATGTTGGTTG | qRT-PCR |
| RBOHeF | TCAAGGCAGCGATTTACCC | qRT-PCR |
| RBOHeR | CTCGCAAGCCTTCCCAAA | qRT-PCR |
| RBOHfF | CTTTCTCCATCACTTCAGCA | qRT-PCR |
| RBOHfR | GGGCCATCTACAAGCAACC | qRT-PCR |
| RBOHgF | GTCAAATGCTTATGCTGTCA | qRT-PCR |
| RBOHgR | TGTCCAGTCTCCGTTTGTT | qRT-PCR |
| RBOHhF | TACTTCGGGCAGACACGGAT | qRT-PCR |
| RBOHhR | GCGGGTTGCTGTCACTAAG | qRT-PCR |
| RBOHiF | ACCTTACCTGCGATTTTCCA | qRT-PCR |
| RBOHiR | ACGAAGCAGTGGTGGGAG | qRT-PCR |
| Osh36F | ATGGCGAAAAGTATGCCCGA | qRT-PCR |
| Osh36R | AGCAGGCAAGTATCCTGGTG | qRT-PCR |
| NYC1F | TGCAAGGACATTGGTTCCGA | qRT-PCR |
| NYC1R | AGAAGCGTGCTCGACTTTCA | qRT-PCR |
| NYC3F | CAGGTGCCAGAATCACCGTA | qRT-PCR |
| NYC3R | ATGGAACTGCAACTGAACCCT | qRT-PCR |
| Osl2F | ACGCTGTTCTTAGCGTGCAA | qRT-PCR |
| Osl2R | CAGTCACCACCAGTTCACCA | qRT-PCR |
| SGRF | AGGGGTGGTACAACAAGCTG | qRT-PCR |
| SGRR  OsGLO1F  OsGLO1R | GCTCCTTGCGGAAGATGTAG  CAGAAGAGCCGAGAGAGCTG  GCTCTTGTCCATCTCTGCGA | qRT-PCR  qRT-PCR  qRT-PCR |
| casHXK1-SF | CGATGACGAGCCTCCGGGAG | Sequence |
| casHXK1-SR | GAACCCAAGCTCCCTCTCGC | Sequence |
