# Supplementary material for: Hexokinase gene OsHXK1 positively regulates leaf senescence in rice
Source: BMC Plant Biol. 2021 Dec 8;21:580. doi: 10.1186/s12870-021-03343-5 (PMC8653616; doi:10.1186/s12870-021-03343-5)
Supplement: Supplementary file 6 — Additional file 6: Table S2. The protein accession numbers used in the phylogenetic analysis. [file 12870_2021_3343_MOESM6_ESM.docx]

**Additional file 6. Table S2. The protein accession numbers used in the phylogenetic analysis**

| Protein | Organism | Accession |
| --- | --- | --- |
| OsHXK1 | *Oryza sativa* | XP_015645221.1 |
| OsHXK2 | *Oryza sativa* | XP_015637797.1 |
| OsHXK3 | *Oryza sativa* | XP_015621344.1 |
| OsHXK4 | *Oryza sativa* | XP_015645316.1 |
| OsHXK5 | *Oryza sativa* | XP_015639323.1 |
| OsHXK6 | *Oryza sativa* | XP_015618116.1 |
| OsHXK7 | *Oryza sativa* | XP_015637554.1 |
| OsHXK8 | *Oryza sativa* | XP_015622018.1 |
| OsHXK9 | *Oryza sativa* | XP_015614778.1 |
| OsHXK10 | *Oryza sativa* | XP_015638932.1 |
| AtHXK1 | *Arabidopsis thaliana* | NP_194642.1 |
| AtHXK2 | *Arabidopsis thaliana* | NP_001077921.1 |
| AtHXK3 | *Arabidopsis thaliana* | NP_175463.1 |
| AtHXK4 | *Arabidopsis thaliana* | NP_188639.2 |
| AtHXK5 | *Arabidopsis thaliana* | NP_175463.1 |
| AtHXK6 | *Arabidopsis thaliana* | NP_195497.1 |
| NtHXK1 | *Nicotiana tomentosiformis* | NP_001312563.1 |
| SlHXK1 | *Solanum lycopersicum* | NP_001233957.1 |
